# Methylphenidate Ameliorates Behavioural and Neurobiological Deficits in Executive Function for Patients with Chronic Traumatic Brain Injury

**DOI:** 10.3390/jcm13030771

**Published:** 2024-01-29

**Authors:** Alexander R. D. Peattie, Anne E. Manktelow, Barbara J. Sahakian, David K. Menon, Emmanuel A. Stamatakis

**Affiliations:** 1 Division of Anaesthesia, University of Cambridge, Addenbrooke’s Hospital, Box 93, Hills Road, Cambridge CB2 0QQ, UK; am807@cam.ac.uk (A.E.M.); dkm13@cam.ac.uk (D.K.M.); 2 Department of Clinical Neurosciences, University of Cambridge, Addenbrooke’s Hospital, Box 165, Hills Road, Cambridge CB2 0QQ, UK; 3 Department of Psychiatry, University of Cambridge, Herchel Smith Building for Brain and Mind Sciences, Forvie Site, Robinson Way, Cambridge CB2 0SZ, UK; bjs1001@cam.ac.uk; 4 Wolfson Brain Imaging Centre, University of Cambridge, Cambridge Biomedical Campus, Box 65, Cambridge CB2 0QQ, UK

**Keywords:** traumatic brain injury, diffuse axonal injury, methylphenidate, executive function, Tower of London, neuroimaging, fMRI, management, rehabilitation

## Abstract

(1) **Background**: Traumatic brain injury (TBI) often results in cognitive impairments, including in visuospatial planning and executive function. Methylphenidate (MPh) demonstrates potential improvements in several cognitive domains in patients with TBI. The Tower of London (TOL) is a visuospatial planning task used to assess executive function. (2) **Methods**: Volunteers with a history of TBI (*n* = 16) participated in a randomised, double-blinded, placebo-controlled, fMRI study to investigate the neurobiological correlates of visuospatial planning and executive function, on and off MPh. (3) **Results**: Healthy controls (HCs) (*n* = 18) and patients on placebo (TBI-placebo) differed significantly in reaction time (*p* < 0.0005) and accuracy (*p* < 0.0001) when considering all task loads, but especially for high cognitive loads for reaction time (*p* < 0.001) and accuracy (*p* < 0.005). Across all task loads, TBI-MPh were more accurate than TBI-placebo (*p* < 0.05) but remained less accurate than HCs (*p* < 0.005). TBI-placebo substantially improved in accuracy with MPh administration (TBI-MPh) to a level statistically comparable to HCs at low (*p* = 0.443) and high (*p* = 0.175) cognitive loads. Further, individual patients that performed slower on placebo at low cognitive loads were faster with MPh (*p* < 0.05), while individual patients that performed less accurately on placebo were more accurate with MPh at both high and low cognitive loads (*p* < 0.005). TBI-placebo showed reduced activity in the bilateral inferior frontal gyri (IFG) and insulae versus HCs. MPh normalised these regional differences. MPh enhanced within-network connectivity (between parietal, striatal, insula, and cerebellar regions) and enhanced beyond-network connectivity (between parietal, thalamic, and cerebellar regions). Finally, individual changes in cerebellar-thalamic (*p* < 0.005) and cerebellar-parietal (*p* < 0.05) connectivity with MPh related to individual changes in accuracy with MPh. (4) **Conclusions**: This work highlights behavioural and neurofunctional differences between HCs and patients with chronic TBI, and that adverse differences may benefit from MPh treatment.

## 1. Introduction

Traumatic brain injury (TBI) contributes to worldwide death and disability more than any other trauma-related injury [1,2]. TBI costs the international economy an estimated USD 400 billion annually, 0.5% of the global economic output [3,4]. Between 64–74 million new cases occur annually around the world, and the number of individuals who live with the consequences of TBI is predicted to rise, given the increasing number of TBI survivors [1,5]. Cognitive deficits appear to be key markers of the transition between independent and severely disabled survival [6], and such disability is responsible for substantial individual and family impacts alongside societal costs. Finding effective treatments to ameliorate cognitive deficits could improve outcome and alleviate costs [7].

Clinical, psychological, and cognitive problems associated with TBI include impaired attention, memory, poor executive function, impulsivity, poor decision-making, and depression [8,9,10,11,12]. Certain deficits are sometimes related to frontal and temporal region damage [10,13,14,15,16,17], and such deficits can be mixed, but patients with TBI can display executive dysfunction despite normal basic attention and memory performance [7]. Whether in isolation or in combination with other deficits, executive dysfunction remains a common and disabling cognitive impairment after TBI.

The Tower of London (TOL) task is a visuospatial planning task, with a strong working memory component, that indicates executive function faculties [18,19,20]. It is used extensively to study planning [19,20,21,22,23,24,25,26,27,28,29,30], an important ability for problem solving and essential to healthy cognition [31]. Better TOL performance is associated with increased frontoparietal activity in healthy individuals [23,32,33]. Prefrontal, parietal, anterior cingulate cortices, and the precuneus are consistently activated and crucial in effectively executing the TOL task [24,25,26,34,35,36]. These areas are key to working memory and planning [37,38,39,40,41]. Additional areas include the dorsolateral prefrontal cortex, superior and inferior frontal gyri (IFG), parietal cortices, and insulae [25,27,42,43,44]. fMRI has been used to investigate the neurobiological correlates of planning and executive dysfunction after TBI [22,45,46,47,48,49]. Rasmussen et al. [34] showed that TBI patients displayed more diffuse, widespread frontal, parietal and occipital activity, increased cingulate and insula activity, and a greater degree of right lateralisation during an fMRI-TOL paradigm versus healthy individuals. 

ADHD-like symptoms can emerge following TBI, and present with cognitive deficits in several domains, including attention, response inhibition, working memory, and executive function [27,50,51,52,53,54,55,56]. Despite the differences in aetiology, stimulant medications traditionally prescribed for ADHD are increasingly applied to ameliorate cognitive deficits after TBI by addressing potential neurotransmitter imbalances [40,57,58,59,60,61,62,63]. Methylphenidate (MPh) has long been prescribed to address the neuropsychiatric sequelae of ADHD [64,65,66,67], and increasingly to treat the functional deficits of TBI [61,62,68,69,70,71,72,73]. MPh is a catecholamine reuptake inhibitor which increases dopamine (DA), noradrenaline (NA), and serotonin (5-HT) levels, especially in frontal and striatal areas [66,74,75,76,77,78]. 

At a subcellular level, synaptic connections, axonal projections, and associated neuro-modulatory mechanisms are perturbed in TBI [79,80,81,82,83]. Dopaminergic circuits are often damaged after TBI due to shearing and rotational forces, and DA-rich areas including the prefrontal cortex and striatum are vulnerable [84,85,86,87,88,89]. These catecholaminergic systems play an important role in executive function, working memory, attention, and response inhibition and mediate neurocognitive systems via cortico-striatal-thalamic-cortical (CSTC) loops [90]. MPh improves working memory and planning in healthy individuals [38,39,91,92,93] and has been shown to improve accuracy and reaction time for working memory and visuospatial attention tasks in TBI patients, and this related to changes in parietal and occipital activation in a perfusion fMRI study [57,59,69]. Wilmott and Ponsford [61] suggest that MPh is more beneficial after the acute phase of injury because cellular processes are more regulated in chronic TBI [59,69,90]. We have previously demonstrated that MPh can modulate motor control, working memory, and response inhibition in patients with chronic TBI [71,72,73], and showed that improved performance in these tasks is associated with changes in functional connectivity (FC) in cognate regions and circuits. 

MPh-driven attenuation of executive dysfunction has been shown, via an idiographic approach, to be more apparent in the chronic phases of TBI [94]. However, an investigation into the benefits of MPh on executive function and the associated mechanisms has not been conducted via a double-blinded, placebo-controlled fMRI study in patients with chronic TBI. Therefore, we conducted an exploratory blood oxygenation level dependent (BOLD) fMRI-task-paradigm with patients with chronic TBI to understand the neuroanatomical substrates for MPh-related improvement in executive function during a visuospatial planning task in patients with chronic TBI with generalised white matter damage and suspected diffuse axonal injury (DAI).

## 2. Materials and Methods

### 2.1. Setting

Two experimental cohorts participated in a randomised, double-blinded, placebo-controlled, crossover magnetic resonance imaging (MRI) study. Both cohorts attended two sessions separated by 2–4 weeks. Radiographers at the Wolfson Brain Imaging Centre (WBIC) (Addenbrooke’s Hospital, Cambridge, UK) acquired functional (fMRI) data while subjects performed a randomised order of various cognitive tasks. All participants were observed to ensure task engagement. These fMRI-task paradigms aim to provide an informed basis for selecting targeted neurocognitive enhancers to address cognitive deficits of TBI. Results have been published already for assessing motor control, working memory, and response inhibition [71,72,73]. This paper focuses on the TOL task to investigate the neurofunctional substrates of healthy, impaired, and improved visuospatial planning and executive function.

### 2.2. Participants

Participants were referred from the Addenbrooke’s Neurosciences Critical Care Unit Follow-Up Clinic, Addenbrooke’s Traumatic Brain Injury Clinic, and Royal London Hospital Intensive Care Unit (Figure S1). Preliminary and post-scanning assessments were conducted at the Wellcome Trust Clinical Research Facility, Addenbrooke’s Hospital. Exclusion criteria included scores of National Adult Reading Test < 70, Mini Mental State Examination < 23, left handedness, history of drug/alcohol abuse, history of psychiatric/neurological disorders, MRI scanning contra-indications, medication affecting cognitive performance or prescribed for depression, any physical handicap preventing completion of testing, and pregnancy or nursing (Figure S1).

Patients with a history of TBI (of all severities), without mass focal lesions on acute CT scans, not included in >3 research studies within a calendar year, were approached to voluntarily participate. Forty-two participants met the study criteria (23 Heathy Controls (HCs) and 19 patients) (Figure S1). Only patients who were at least six-months post-TBI (i.e., chronic TBI [95,96]), without mass focal lesions, and suspected DAI were included in this study. Two patients were unable to complete the scanning process, and another had necessary behavioural data missing. The HC volunteers were recruited through local advertisements in the Cambridge area and paid for participation. Three HCs had missing fMRI data, and two missing structural MRI data. Subsequently, these subjects were removed from further analyses. Thereafter, HCs consisted of 12 males and 6 females. Patients consisted of 12 males and 4 females. HCs were aged 19–57 years (mean 34.1 ± 10.7 SD; median 30.5 ± 12.0 IQR). Patients were aged 19–58 years (mean 35.0 ± 14.1 SD; median 31.5 ± 26.5 IQR). Patients (Table 1) did not differ in age from HCs (*p* = 0.830, two-sample *t*-test). Mean duration from injury to first scan was 21.3 months ± 12.2 SD; median 21.0 months ± 22.25, for TBI patients.

During their first visits and before receiving medication, background assessments were performed for patients and HCs, lasting ~30 min. These included questionnaires assessing current psychiatric status and quality of life (e.g., the Neuropsychiatric Inventory (patients only), Beck’s Depression Inventory, SF-36, Glasgow Outcome Scale (Extended) (patients only)) (Figure S2; Table S1). A baseline cognitive assessment was conducted using the Cambridge Neuropsychological Test Automated Battery (CANTAB) system (©Cambridge Cognition; Cambridge, Cambridgeshire, UK). Tests assessed working memory capacity (spatial span: SSP), episodic memory (paired associated learning: PAL), executive function (intra/extradimensional set shift: IED), and simple reaction time (SRT) (Figure S3; Table S2).

The Cambridgeshire 2 Research Ethics Committee approved the study (LREC 08/H0308/246). It was conducted according to Good Clinical Practice Guidelines, Declaration of Helsinki, and the United Kingdom Central Council Code of Conduct. All participants provided written informed consent before participation.

### 2.3. Experimental Design

HCs received no pharmacological intervention. TBI patients were randomly allocated, via a Latin Square design, to receive one of two visually indistinguishable medications on their first visit, with the other on their second visit. These medications contained either a 30 mg Methylphenidate (MPh) tablet or a placebo tablet containing lactose. Thirty milligrams MPh was given to ensure an effective dose was administered whilst minimising the likelihood of side-effects, and in keeping with protocols in previous studies [97,98,99,100] and adult medication guidelines from the UK National Institute for Health and Care Excellence (NICE; which recommends a dose of 15–100 mg). After a 75 min delay to ensure peak plasma levels were reached, patients completed a 45–60 min fMRI scan with both resting-state fMRI (rs-fMRI) and task-based fMRI, using a randomised task order (Figure S1).

### 2.4. Tower of London Task Stimulus Presentation

The fMRI-TOL task used a randomised trial design consisting of five conditions: *Count*, *1 move*, *2 moves*, *3 moves*, and *4 moves*, each consisting of 10 trials and inter-dispersed with *fixation* conditions. During count blocks, participants aimed to count the number of balls on the viewable scanner screen (Figure S4). During move blocks, participants were asked to estimate the minimum number of moves required from a start configuration to match the target configuration. During *fixation* blocks between trials, participants fixated their gaze on a white cross at the screen centre. Trial stimulus onset time, reaction time, and response outcome during blocks were recorded for each participant (Supplementary Notes).

### 2.5. MRI Data Acquisition and Presentation

MRI data were acquired in a Siemens Trio-3Tesla MR system (Siemens AG, Munich, Germany). For each participant, localiser images and 3D high-resolution MPRAGE images (Relaxation Time (TR) = 2300 ms, Echo Time (TE) = 2.98 ms, Flip Angle = 9°, FOV = 256 mm^2^ × 256 mm^2^) were acquired for anatomically informed pre-processing of functional scans to spatially normalise to Montreal Neurological Institute (MNI) space. Neuroradiologists reviewed all single T1-weighted structural scans to confirm all patients presented without substantial mass lesions and with suspected DAI. An echo-planar imaging (EPI) sequence acquired fMRI data with parameters TR = 2000 ms, TE = 30 ms, Flip Angle = 78°, FOV = 192 mm^2^ × 192 mm^2^, 32 slices 3.0 mm thick with a gap of 0.75 mm between slices.

### 2.6. fMRI Data Pre-Processing

Statistical Parametric Mapping (SPM) software, Version 12 (Wellcome Department of Cognitive Neurology, (https://www.fil.ion.ucl.ac.uk/spm/) implemented in MATLAB 2016a (https://www.mathworks.co.uk/products/matlab/; Mathworks, Sherborn, MA, USA) pre-processed and analysed fMRI data. Functional image pre-processing first removed the first five volumes for each participant to control for initial signal instability. Subsequent image pre-processing included slice-timing correction, within-participant realignment, movement correction, structural scan co-registration to the mean fMRI-EPI image, co-registered structural image segmentation, and derivation of spatially normalised grey matter probabilistic images. The spatial normalisation parameters obtained were applied to realigned fMRI images to bring them into MNI space. Functional images were smoothed using an isotropic 6 mm full-width half-maximal (FWHM) Gaussian kernel to minimise residual anatomical differences and increase the signal-to-noise ratio.

### 2.7. Behavioural Data Analyses

Behavioural data analyses were performed using RStudio Version 2023.09.0 + 463 for MacOS (©2009–2023 RStudio, Inc., Boston, MA, USA). Accuracy and reaction time performance measures were investigated during the TOL task, comparable to previous studies [101]. We focused on the *Count*, *1 move*, and *3 moves* conditions to respectively assess differences in basic cognition, and low and high visuospatial planning ability as an indication of executive function abilities [102]. These conditions were chosen in accordance with ‘load theory’, i.e., the ability to focus improves with conditions of high perceptual loads, but not so high as to deteriorate with excessive cognitive control processes [103,104,105,106,107,108], a phenomenon more pronounced after TBI [108,109]. Mean reaction times and accuracy are reported for each condition for each group: HCs, TBI-MPh, and TBI-placebo. Wilcoxon rank-sum tests were used for two-sample group comparisons between HCs and TBI-placebo as well as HCs and TBI-MPh. Wilcoxon signed-rank tests were used for paired-sample group comparisons between TBI-MPh and TBI-placebo. Spearman’s correlations were adopted to investigate the relationship between changes in performance when comparing the TBI-placebo visits to the change in performance with MPh between the TBI-MPh and TBI-placebo visits (MPh minus placebo) for individual subjects.

### 2.8. fMRI Data Analyses and Statistical Modelling

Pre-processed functional images for each participant entered a voxel-based model, within a first-level general linear model (GLM) framework. This GLM was based on the randomised presentation for all stimulus types (*Count*, *1 move*, *2 moves*, *3 moves*, *4 moves*), and generated by modelling all trial stimulus onset times, response times, computed reaction times, and haemodynamic response function. This was done so the model was not underfitted for this exploratory analysis. Six movement parameters were included as regressors to minimise false positives linked to residual movement artefacts. A high-pass filter with a period of 128 s removed low-frequency scanner noise. 

We implemented subtractive analyses to reveal brain regions and networks which were essential to visuospatial planning compromised due to chronic TBI and potentially improved with MPh. Subtractive analyses enabled activation measured from baseline *fixation* blocks to be subtracted from activation measured at task. Resulting contrast images were entered into a second-level group analysis involving one-sample *t*-tests to obtain within-group brain activations for each group: HCs, TBI-placebo, and TBI-MPh. Group differences between HCs and patient cohorts used two-sample *t*-tests. For comparisons within patient cohorts at placebo and MPh visits, we used paired-sample *t*-tests. Results of analyses were considered significant if *p* ≤ 0.001 (uncorrected) at the voxel level and *p* ≤ 0.05 (corrected for multiple comparisons, Family-Wise Error correction, FWEc) at the cluster level. The significant peak co-ordinates from activity analyses were annotated using MRIcroN software and the ICN atlas toolbox [110]. Investigating brain activation involved comparing conditions by focusing on *Count* to assess basic computation, *1 move* and *3 moves* to respectively assess lower and higher cognitive loads, and *fixation* for visual input. The contrasts examined were *1 move* > *Count*, *3 moves* > *Count*, *Count* > *fixation*, *1 move* > *fixation*, and *3 moves* > *fixation* to compare activations during the task and investigate which brain regions contribute to healthy, compromised, and improved executive function and planning during the TOL task.

### 2.9. Task-Modulated Functional Connectivity Analyses

Twenty-three regions of interest (ROIs) were defined by constructing a 6 mm sphere around peaks identified by the subtractive activation analyses, and BOLD task-specific timeseries were extracted for each of these (Figure S6). Broadly, these were frontoparietal, limbic, subcortical, and cerebellar regions. We call this the visuospatial planning network and examined its function at the higher cognitive load (*3 moves* > *fixation*) to reveal how significant regions were coordinated at task, perturbed in TBI, and modulated with MPh. This cognitive load choice was informed by behavioural- and activity-related analyses. At the high load, the maximum effect of the task, impairments of chronic TBI, and potential benefit of MPh should be clearly reflected in these exploratory connectivity analyses.

Furthermore, we used Generalised Psychophysiological Interaction (gPPI) and whole brain voxel-wise Psychophysiological Interaction (PPI) analyses to investigate the task-modulated functional connectivity (FC) within and beyond networks. PPI frameworks are useful for investigating how activity in one brain region relates to activity in other brain areas when undertaking specific tasks [111]. The first-level GLM included a task-specific timeseries for each subject, specifically the *3 moves* > *fixation* contrast, and related regressors. The second-level analyses considered both within-group brain connectivity patterns for each group (using one-sample *t*-tests), and between-group brain connectivity differences from the subtractive analyses (paired- and two-sample *t*-tests). These analyses enabled us to explore what connectivity contributed to healthy, compromised, and pharmacologically improved executive function and planning during the TOL task. Results of analyses were considered significant if *p* ≤ 0.001 (uncorrected) at the voxel level and *p* ≤ 0.05 (FWEc) at the cluster level. The significant peak co-ordinates and the spatial extent of the cluster from connectivity analyses were annotated using MRIcroN software and the ICN atlas toolbox [110].

### 2.10. Behavioural–Functional Correlations

We explored whether changes in connectivity of specific ROIs was related to changes in behaviour under MPh administration at *3 moves* > *fixation*. Patient-specific ROI–ROI gPPI correlations were extracted for TBI-MPh > TBI-placebo. Spearman’s correlations were adopted to investigate the relationship between individual subjects’ changes in performance when comparing the TBI-MPh and TBI-placebo visits to the change in connectivity when looking at the TBI-MPh > TBI-placebo contrast comparison. This aimed to identify a neurofunctional marker for MPh-modulated executive function in patients with chronic TBI.

## 3. Results

### 3.1. Behavioural Differences

#### 3.1.1. Differences in Mean Reaction Time

**Figure 1:**
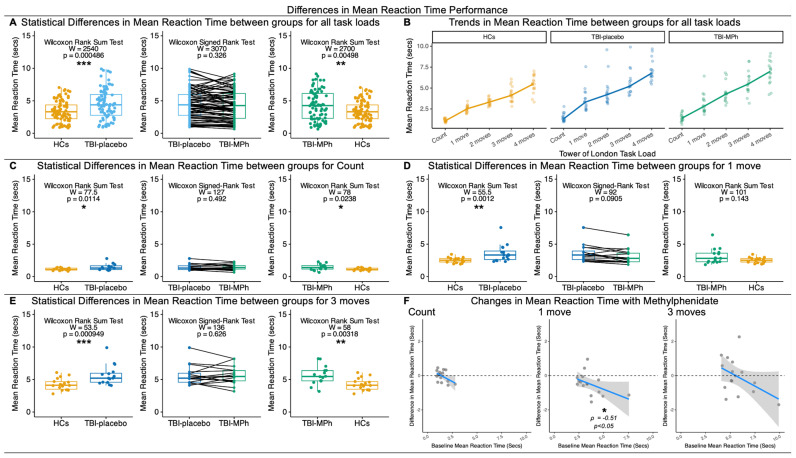
Behavioural Differences in Mean Reaction Time (s) during a Tower of London task for Healthy Controls (HCs), Patients with chronic TBI on placebo (TBI-placebo), and Patients with chronic TBI on Methylphenidate (TBI-MPh). (**A**) Statistical Differences in Mean Reaction Time between groups for all task loads: Wilcoxon Rank Sum Tests indicate that TBI-placebo and TBI-MPh have a slower reaction time than HCs across all task loads. (**B**) Trends in Mean Reaction Time in HCs, TBI-placebo, and TBI-MPh across all task loads. TBI-MPh ameliorates deficits in reaction time at *1 move*. (**C**) Statistical Differences in Mean Reaction Time between groups for *Count*. Wilcoxon Rank Sum Tests indicate that TBI-placebo and TBI-MPh have a slower reaction time than HCs for *Count*. (**D**) Statistical Differences in Mean Reaction Time between groups for *1 move*. Wilcoxon Rank Sum Tests indicate that TBI-placebo have a slower reaction time than HCs for *1 move*. (**E**) Statistical Differences in Mean Reaction Time between groups for *3 moves*. Wilcoxon Rank Sum Tests indicate that TBI-placebo and TBI-MPh have a slower reaction time than HCs for *3 moves*. (**F**) Spearman’s correlations between change in Mean Reaction Time (TBI-MPh—TBI-placebo) versus baseline Mean Reaction Time (TBI-placebo). Spearman’s rho (ρ) indicates Methylphenidate ameliorates deficits in Mean Reaction Time at *1 move*. Dashed line indicates zero, and dotted line indicates a ceiling effect. ns: *p* > 0.05; *: *p* < 0.05; **: *p* < 0.01; ***: *p* < 0.001.

Behavioural differences in mean reaction time were analysed for HCs, TBI-placebo, and TBI-MPh (Figure 1). Across the whole task, patients with chronic TBI, whether they were on or off MPh, demonstrated deficits in mean reaction time compared to HCs across all task loads (Figure 1A). Yet, when splitting the task according to task load, potential differences between TBI-placebo and TBI-MPh were observed at *1 move* (Figure 1D). Indeed, HCs were faster than TBI-placebo for *Count* (Figure 1C), *1 move* (Figure 1D), and *3 moves* (Figure 1E). HCs were faster than TBI-MPh for *Count* (Figure 1C) and *3 moves* (Figure 1E). This indicates that MPh combats reaction time deficits at low cognitive loads for *1 move*. Indeed, certain patients that performed more slowly on placebo performed faster with MPh at *1 move* (Figure 1F), perhaps contributing to changes in mean reaction time with MPh treatment at low cognitive loads.

#### 3.1.2. Differences in Accuracy

**Figure 2:**
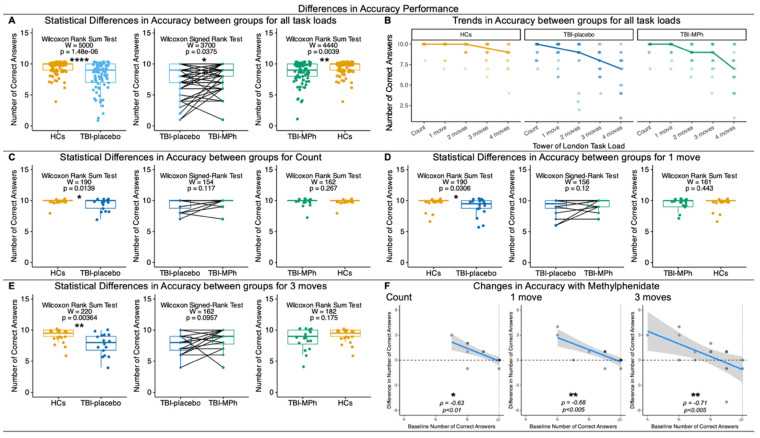
Behavioural Differences in Accuracy during a Tower of London task for Healthy Controls (HCs), Patients with chronic TBI on placebo (TBI-placebo), and Patients with chronic TBI on Methylphenidate (TBI-MPh). (**A**) Statistical Differences in Accuracy between groups for all task loads: Wilcoxon Rank Sum Tests indicate that TBI-placebo and TBI-MPh are less accurate than HCs across all task loads and TBI-MPh are more accurate than TBI-placebo across all task loads. (**B**) Trends in Accuracy in HCs, TBI-placebo, and TBI-MPh across all task loads. TBI-MPh ameliorates deficits in accuracy at *1 move* and *3 moves*. (**C**) Statistical Differences in Accuracy between groups for *Count*. Wilcoxon Rank Sum Tests indicate that TBI-placebo have a lower accuracy than HCs for *Count*. (**D**) Statistical Differences in Accuracy between groups for *1 move*. Wilcoxon Rank Sum Tests indicate that TBI-placebo have a lower accuracy than HCs for *1 move*. (**E**) Statistical Differences in Accuracy between groups for *3 moves*. Wilcoxon Rank Sum Tests indicate that TBI-placebo have a lower accuracy than HCs for *3 moves*. (**F**) Spearman’s correlations between change in accuracy (TBI-MPh—TBI-placebo) versus baseline accuracy (TBI-placebo). Spearman’s rho (ρ) indicates Methylphenidate ameliorates deficits in Accuracy at *Count*, *1 move*, and *3 moves*. Dashed line indicates zero, and dotted line indicates a ceiling effect. ns: *p* > 0.05; *: *p* < 0.05; **: *p* < 0.01; ****: *p* < 0.0001.

Behavioural differences in accuracy were analysed for HCs, TBI-placebo, and TBI-MPh (Figure 2). Across all task loads, both TBI-placebo and TBI-MPh were less accurate when compared to HCs (Figure 2A). However, TBI-MPh were marginally more accurate than TBI-placebo (Figure 2A), contrary to differences in mean reaction time (Figure 1A). Across all task loads, TBI-MPh were marginally more accurate than TBI-placebo (Figure 2A). When splitting the task according to task loads, HCs were faster across all task loads compared to TBI-placebo, while HCs were more accurate across *2 moves* and *4 moves* task loads compared to TBI-MPh ((Figure 2B). Undeniably, HCs were more accurate than TBI-placebo for *Count* (Figure 2C), *1 move* (Figure 2D), and *3 moves* (Figure 2E). HCs and TBI-MPh were not different in accuracy for *Count* (Figure 2C), *1 move* (Figure 2D), and *3 moves* from TBI-placebo (Figure 2E). Yet, differences between HCs and TBI-MPh were not observed at *Count* (Figure 2C), *1 move* (Figure 2D), and *3 moves* (Figure 2E). This indicates that MPh improves accuracy at low and high cognitive loads for *Count*, *1 move*, and *3 moves*. Across these task loads, patients that were less accurate on placebo were more accurate on MPh at *Count*, *1 move*, and *3 moves* (Figure 2F), suggesting a role for MPh in ameliorating executive dysfunction, especially at *1 move* and *3 moves*.

### 3.2. Activity Differences

#### Activity Differences Associated with Visuospatial Planning in TBI and with MPh

**Figure 3:**
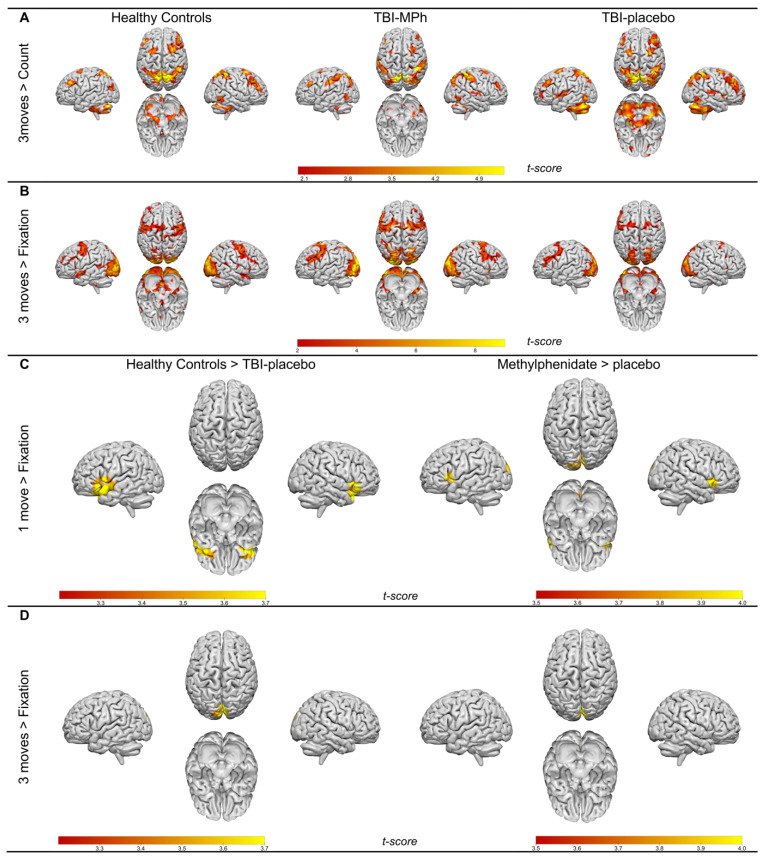
Differences in activity for the *3 moves* versus *Count*, the *1 move* versus *fixation*, and the *3 moves* versus *fixation* contrasts. (**A**) Significant activation peaks for the *3 moves* versus *Count* contrast for each experimental group. (**B**) Significant activation peaks for the *3 moves* versus *fixation* contrast for each experimental group. (**C**) Statistically significant activation peaks for the *1 move* versus *fixation* contrast for HCs and TBI-MPh versus TBI-placebo. (**D**) Statistically significant activation peaks for the *3 moves* versus *fixation* contrast for HCs and TBI-MPh versus TBI-placebo. No significant differences between HCs and TBI-MPh. Results are superimposed on a template supplied by Surf Ice (https://www.nitrc.org/projects/surfice/).

During the TOL task, HCs, TBI-placebo, and TBI-MPh all exhibited frontoparietal, subcortical, and occipital activations when comparing task versus baseline contrasts (Figure 3; Table S3). To investigate regions exclusive for visuospatial planning, we focused on the *moves* > *count* contrasts, and demonstrated activation in a frontoparietal network with subcortical and cerebellar contributions (Figure 3A; Table S3). While the *3 moves* > *Count* condition demonstrated a greater right lateralisation between TBI and HCs, we examined the *moves* > *fixation* contrast to provide a more complete picture of processing differences between TBI and HCs, and found that patients displayed activity in parietal, posterior-frontal, and subcortical regions (Figure 3B; Table S3).

HCs and TBI-placebo groups showed activity differences at both low and high cognitive loads. For the *1 move* > *fixation* comparison, we found differences between HC and TBI-placebo in the bilateral orbital IFG, superior temporal poles, and right insula (Figure 3C; Table S4). At the higher cognitive load, a similar comparison revealed activity differences for *3 moves* > *fixation* in occipital regions, including the bilateral cuneus, calcarine sulci, and left superior occipital gyrus (Figure 3D; Table S4). Two-sample *t*-tests at *1 move* > *Count* and *3 moves* > *Count* revealed no statistically significant differences in activity when comparing HCs and TBI-placebo.

Paired-sample *t*-tests between TBI-MPh and TBI-placebo found activity differences for *1 move* > *fixation* in the bilateral insulae, cuneus, lingual gyri, calcarine sulci, and right opercular IFG (Figure 3C; Table S3). At the higher cognitive load, *3 moves* > *fixation* differences were situated in occipital regions including bilateral cuneus, calcarine sulci, and the right superior and middle occipital gyri (Figure 3D; Table S3). Two-sample *t*-tests at *1 move* > *Count* and *3 moves* > *Count* did not reveal statistically different activation between TBI-MPh and TBI-placebo. We did not find statistically significant activity differences between HCs and TBI-MPh at *1 move* > *Count*, *3 moves* > *Count*, *1 move* > *fixation*, and *3 moves* > *fixation*. This suggested a role for MPh in normalising functional differences associated with chronic TBI.

Overall, MPh normalised, at least partially, activation differences between HCs and TBI-placebo at both high and low cognitive loads. The shared upregulated activity in HCs and TBI-MPh versus TBI-placebo were in the IFG, bilateral superior temporal poles, right insula, and occipital regions. MPh, therefore, ameliorated activity differences between HCs and TBI-placebo at cognitive loads where MPh ameliorated behavioural differences between HCs and TBI-placebo.

### 3.3. Connectivity Differences

#### 3.3.1. Within-Network Task-Modulated Functional Connectivity

**Figure 4:**
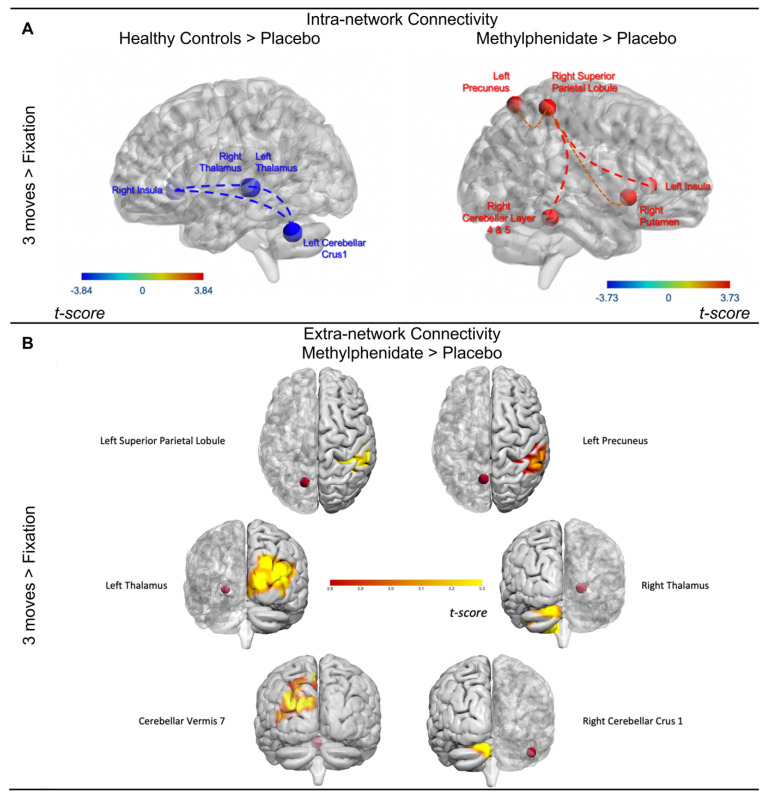
Statistically significant differences in intra-network and extra-network connectivity. (**A**) Significant differences in gPPI, i.e., task-modulated connectivity relationships for the *3 moves* versus *fixation* contrast for HCs versus TBI-placebo (left) and TBI-MPh versus TBI-placebo (right). Red indicates correlations, and blue indicates anti-correlations for the group-contrast in question, respectively. No significant differences between HCs and TBI-MPh. (**B**) Significant differences in task-modulated functional connectivity (PPI) in patients with TBI on Methylphenidate versus Placebo for six regions of interest (red spheres). Results are superimposed on a template supplied by Surf Ice (https://www.nitrc.org/projects/surfice/) and BrainNet Viewer (Xia et al., [119]) (https://www.nitrc.org/projects/bnv/).

Chronic TBI patients have persistent compromised structural and functional connectivity due to grey and white matter damage [112,113,114,115,116,117,118]. This was investigated initially within a network formed by a set determined to be active during the execution of the task, specifically the *3 moves* > *fixation* contrast. TBI-placebo displayed heightened connectivity between the right insula, bilateral thalamus, and left cerebellum versus HCs (Figure 4A). TBI-MPh displayed enhanced positive connectivity above and beyond placebo in the network important for visuospatial planning between the right superior parietal lobule and primarily the left insula and left cerebellum as well as the left precuneus, and right putamen (Figure 4A). HCs and TBI-MPh did not display differences in connectivity between the regions composing this network. This suggests MPh normalised connectivity differences between TBI-placebo and HCs within this network. 

#### 3.3.2. Whole-Brain Voxel-Wise Psychophysiological Interaction Analyses

At the higher cognitive load (*3 moves* > *fixation*), TBI-placebo had connectivity deficits versus HCs, including from the left opercular IFG, left cerebellar crus 1, and cerebellar vermi to additional frontal and temporal regions (Figure S7; Table S6). TBI-MPh displayed an upregulated positive connectivity from the left thalamus to occipital regions versus HCs (Figure S8; Table S6). TBI-MPh demonstrated upregulated connectivity between the left superior parietal lobule and right supramarginal gyrus over TBI-placebo (*p* < 0.001) as well as increased connectivity between the left precuneus and right supramarginal gyrus, left thalamus and right middle occipital gyrus, and left calcarine cortex (*p* < 0.005) (Figure 4B; Table S5). TBI-MPh showed upregulated connectivity from the cerebellum to the left angular gyrus and left inferior parietal lobule versus TBI-placebo (Figure S8; Table S6). Higher connectivity with MPh was found between the right thalamus and cerebellar regions with increases in *intra*-cerebellar connectivity, and lastly, between the cerebellar vermi and occipital regions (Figure 4B; Table S5). This indicates the neurofunctional significance for parietal, thalamic, and cerebellar connectivity in patients with chronic TBI and administration of MPh during the high cognitive loads of a visuospatial planning task that assesses working memory and executive function.

### 3.4. Functional Brain–Behaviour Relationships

**Figure 5:**
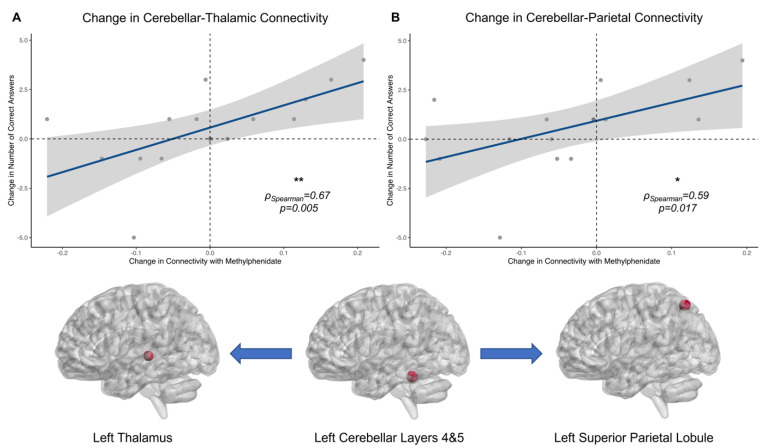
Behavioural–functional relationships between changes in cerebellar-thalamic, changes in cerebellar-parietal connectivity, and changes in accuracy with Methylphenidate. (**A**) Spearman’s correlations between change in accuracy (TBI-MPh—TBI-placebo) and change in connectivity between the cerebellar and thalamic regions of interest (red spheres) during the *3 moves* > *fixation* contrast. (**B**) Spearman’s correlations between change in accuracy (TBI-MPh—TBI-placebo) and change in connectivity between the cerebellar and parietal regions of interest (red spheres) during the *3 moves* > *fixation* contrast. ns not significant; * Significant at *p* < 0.05; ** Significant at *p* < 0.005.

Informed by the within-network and beyond-network connectivity and motivated by our group’s previous work, we investigated whether changes in connectivity between TBI-MPh and TBI-placebo related to changes in accuracy between MPh and placebo at the *3 moves* cognitive load. We demonstrated that individual connectivity changes with MPh administration between the cerebellum and the thalamus (Figure 5A) (log_e_(S) = 5.43, *p* = 0.005, ρ_Spearman_ = 0.67, CI_95%_[0.24, 0.88], n_pairs_ = 16) and the parietal lobule (Figure 5B) (log_e_(S) = 5.64, *p* = 0.017, ρ_Spearman_ = 0.59, CI_95%_[0.11, 0.84], n_pairs_ = 16) positively correlate with changes in accuracy with MPh. This highlights an important relationship between changes in thalamic-cerebellar and parietal-cerebellar connectivity and improved performance of MPh in ameliorating neurocognitive deficits in patients with chronic TBI [120,121,122,123,124].

## 4. Discussion

This exploratory study investigated the effects of MPh on patients with chronic TBI during a visuospatial planning task. Broadly, TBI-placebo displayed behavioural and neurobiological deficits marked by poorer performance for reaction time and accuracy alongside reduced activity and connectivity in regions associated with a visuospatial planning task. Statistically significant activity differences were found in the insulae, IFG, and occipital cortex. Similarly, significant differences in connectivity were found in the IFG, insulae, thalamus, parietal cortex, and cerebellum. These regions have previously been implicated in the TOL task and recruited in neuropsychologically compromised individuals or during challenging cognitive tasks in healthy individuals. Importantly, MPh administration normalised aspects of behavioural performance alongside differences in activity and connectivity. HCs and TBI-MPh displayed similar activity differences compared to TBI-placebo. Both TBI-MPh and HCs displayed heightened connectivity between parietal, subcortical, occipital, and cerebellar regions versus TBI-placebo. TBI-MPh displayed connectivity above and beyond HCs in the thalamus, perhaps revealing part of the mechanism behind behavioural improvement with this stimulant medication. Notably, this study included only patients with chronic TBI with no mass lesions and suspected DAI. Therefore, differences seen related to diffuse connectivity rather than focal excisions. The overall proposition is that MPh has a beneficial use in this cognitive domain for this cohort, as our group has demonstrated in other cognitive domains with previous work [71,72,73].

Aligned with previous research, HCs produced faster and more correct responses than TBI patients according to reaction time and accuracy [69,71,72,73,95,101,125,126]. TBI-MPh were more accurate across the whole task but not faster than TBI-placebo. Most notably, deficits in accuracy between HCs and TBI-placebo were minimised with MPh at high cognitive loads. Similarly, Kim et al. [69] noted no decrease in reaction time but significant improvements in response accuracy in the MPh group for a visuospatial attention task versus a working memory task in their TBI cohort and suggested these two tasks are subserved by different circuits. 

Predictive markers of whether specific treatments might aid individuals are advantageous for patient care. Simple measures (e.g., age at scan, time since injury, GCS and GOSE) did not predict performance enhancement within this cohort of patients with chronic TBI. However, exploring accuracy enhancement demonstrated that poorer patient performance on placebo potentially benefits more from MPh administration—as our group’s earlier studies have demonstrated [71,72,73]. Contrary to other research on MPh’s cognitive effects, we noted benefits that were more pronounced for accuracy versus reaction time for an executive function task [57,59,61,62,63,68,69]. 

Since TBI-related damage is often to vulnerable frontal and temporal regions, we expected attenuated frontal, temporal, and occipital activity together with performance deficits in this TBI cohort [34]. We found activity differences between HCs and TBI-placebo in such areas, including the IFG and insulae. Comparably, activity differences between TBI-MPh and TBI-placebo were found in regions similar to those where differences between HCs and TBI-placebo were found, including the IFG and insulae. This strengthened the assertion that the IFG and insulae are important for both healthy and improved visuospatial planning and working memory. The IFG has long been implicated in working memory [127,128,129], and working memory is necessary for successful performance during the TOL task [21,130,131,132,133,134,135]. Further, activity in the adjacent anterior insula was observed in healthy individuals during higher cognitive loads in the TOL task [27,34,36,136]. Additionally, neuroanatomical and functional alterations of the insula have been found with TBI [137]. These areas are important for healthy visuospatial manipulation and directed attention, and tasks of high-level attention, perception, and control [138,139,140]. The anatomical basis for this connected cognitive role is thought to be the insula’s extensive structural connections to multiple regions, including the orbitofrontal cortex [141] (Figure S5). Indeed, MPh-medicated TBI and ADHD patients displayed significant additional activations, including in the insulae and IFG during tasks like this executive function task [71,72,73,137]. 

However, MPh primarily acts on the basal ganglia, including the dorsal striatum and caudate [66,74,75,76,77] (Figure S5). These subcortical regions are important for numerous attention-demanding cognitive functions [142,143,144,145]. The insula is well-connected to these areas and the thalamus [146,147,148] (Figure S5). It is well-positioned to mediate networks of cognitive domains, including executive function, working memory, attention, and goal-directed behaviour [149]. Critically, the IFG are reciprocally connected to superior frontal regions (Figure S5; Figure S7) and inferior and superior parietal areas crucial to the planning network, i.e., the frontoparietal network [27,150,151] (Figure S5). Frontoparietal regions are involved in working memory, visuospatial planning, and executive function [152,153].

Examining how connectivity is perturbed in TBI is crucial for understanding how cognitive function is compromised with TBI in domains including visuospatial planning, working memory, and executive function [86,96,106,116,154,155,156,157,158,159,160,161,162]. TBI and especially DAI are considered disorders of brain connectivity, which in turn lead to inefficient functional networks and neurocognitive dysfunction [48,49,115]. Accordingly, HCs displayed higher connectivity between the inferior and superior frontal gyri, alongside cerebellar regions and temporal areas, when compared to TBI-placebo (Figure S7). This reiterates the detrimental effects of frontal and temporal damage while highlighting the cerebellum’s importance in effective TOL task execution and executive function [10,13,14,15,16,17,36,163,164,165]. Similarly, investigating how FC is modulated by MPh provided insights into how functional networks may be reconfigured in chronic TBI. TBI-MPh displayed enhanced within-network connectivity above and beyond TBI-placebo between insula, parietal, subcortical, and cerebellar regions. Additionally, TBI-MPh displayed enhanced contralateral connectivity between left parietal areas and the right supramarginal gyrus. These parietal areas are important for working memory and computational processing, which increases FC after TBI [137,166,167,168,169]. These regions may be employed in TBI patients as a response to MPh administration.

These findings support the importance of regions including the striatum, thalamus, insula, and IFG needed for coordinated neurobiological function directed towards executive function, working memory and visuospatial planning in patients with chronic TBI [38,39,59,170,171]. TBI-MPh may be utilising areas less vulnerable to and more cushioned from TBI, including the IFG and insulae, to enhance connectivity through the striatum and thalamus to posterior frontoparietal regions and the cerebellum to ameliorate executive dysfunction associated with chronic TBI [72,73,172] (Figure S5).

Again, MPh mainly affects subcortical nuclei including the putamen and caudate, which form the dorsal striatum [77] (Figure S5). These form parts of the basal ganglia and have much higher presynaptic DA, NA, and 5-HT transporter densities versus the cerebellum, parietal cortex, and frontal regions [74,75,76]. These subcortical regions are DA-rich areas and considered important for reward- and goal-directed behaviour [173,174]. They are often dysfunctional in TBI and other neuropsychiatric conditions [175,176]. Therefore, the dorsal striatum is a primary candidate for dopaminergic modulation through MPh administration [66,74,75,76,77]. These dopaminergic circuits are thought, in turn, to modulate behaviour via CSTC loops, and their DAT levels may predict beneficial MPh treatment [177,178] (Figure S5). Due to TBI’s heterogeneity, it may be even more useful to assess the patient’s dopaminergic deficits via PET or DTI before administering MPh [87,88]. Considering the improved connectivity with MPh between the putamen and parietal regions during this TOL task, we suggest the anatomical basis for increases lies in multiple afferent dopaminergic CSTC loops [178,179,180,181].

CSTC loops critically influence activity within the prefrontal cortex, thought to be heavily involved in domains like executive function [182,183]. The prefrontal cortex is entrenched in additional cortico-cortical loops, i.e., frontoparietal areas, which are crucial for working memory and visuospatial planning [25,153,184]. We have shown that MPh upregulated parietal connectivity. Additionally, we have demonstrated that MPh enhanced connectivity in limbic, thalamic, and cerebellar regions. Enhanced connectivity with MPh was seen from thalamic regions to occipital and cerebellar regions, from cerebellar to parietal and occipital regions, and heightened *intra*-cerebellar connectivity. Indeed, MPh-induced connectivity changes between the cerebellum, thalamus, and superior parietal lobule are related to changes in accuracy with MPh (Figure 5). These results demonstrate the importance of parietal-cerebellar and thalamic-cerebellar connectivity and coordination for visuospatial planning and working memory in chronic TBI patients. 

Previous studies on healthy participants have found activation in the cerebellum and insula alongside an expected frontoparietal activation at high and low cognitive loads [21,33,36,59,163]. This study has also revealed a significant role for the cerebellum at the high cognitive load in the TOL task. Heightened connectivity was shown in HCs and TBI-MPh over TBI-placebo from cerebellar regions to temporal and parietal areas, respectively. For TBI-MPh, this included regions such as the superior parietal lobule and angular gyrus and from the thalamus to cerebellar regions, in addition to heightened *intra*-cerebellar connectivity. Previous TOL imaging studies have demonstrated the cerebellum’s significance in various cognitive processes, including visuospatial planning and working memory [36,164,185]. It is suggested that cerebellar areas such as lobules VI, crus I and VIIB are “topographically” dedicated to executive function [186,187,188,189]. We found that the right thalamus was effectively connected to multiple cerebellar regions, including the left cerebellar crus 1 and left cerebellar lobule IX. We also found a relationship with both the thalamus’s and superior parietal lobule’s connectivity with the cerebellum layer 4&5 related to improved performance with MPh (Figure 5). 

The thalamus is important for routing and coordinating cerebral-cerebellar connectivity for cognitive function, especially considering that infarcts to the thalamus lead to deficits in memory, executive function, and attention [190,191]. The thalamus is a hub that has important anatomical connections to additional subcortical, cortical, and cerebellar regions [124,192,193]. Connectivity deficits between frontoparietal regions, thalamus, and cerebellar regions help explain cognitive deficits in executive function in patients with TBI, especially since the cerebellum integrates visual sensory information, feeds it forward to the thalamus, and on to the frontoparietal regions for higher-level cognitive processing [122,123,124]. This connectivity is a potential secondary target for neuromodulation, as it is in ADHD via fronto-striatal-cerebellar circuits and CSTC loops [2,53,87,194,195].

A study by Allen et al. [196] found that the cerebellum acts as an “error-correction system” during working memory [120,121] and is functionally connected to regions crucial for a network dedicated to working memory [122]. This may be what contributes to accuracy and sensitivity measures, just as previous work from our group has found [72]. These weakened connections in patients with TBI may be enhanced by MPh (Figure 5). Some of these patients had upregulated cerebellar-thalamic and cerebellar-parietal connectivity with MPh that related to improvements in accuracy with MPh. This suggests that the cerebellum may play a role in error correction and accuracy and may contribute to feedback loops within these networks. MPh may provide a catecholaminergic boost that facilitates enhancing connections in cerebellum-thalamus (i.e., CSTC) and cerebellum-basal ganglia (fronto-striatal-cerebellar) loops, thus explaining how patients with moderate cognitive deficits may benefit from it [72]. However, MPh’s benefit may depend on the severity of structural (connectivity) damage in a network dedicated to working memory, visuospatial planning, and executive function [71,72,73], since *severe* TBI cases might lack enough residual structural connectivity to respond to treatment. Perhaps it is beneficial to stratify patients with TBI based on the severity of their structural damage and cognitive dysfunction in order to improve the effectiveness of MPh treatment. Further research is needed to validate this approach.

A key limitation of this study was the inhomogeneity amongst the participants. The mechanisms, the location, time since, and the severity of the trauma varied across the cohort (Table 1). The heterogeneity of this small cohort perhaps makes generalising findings difficult. A larger number of patients with more similar injuries, in terms of severity and location, would provide more concrete conclusions. Regardless, this work is comparable to previous studies, provides a good pilot basis to inform future work, and suggests MPh is useful for ameliorating executive dysfunction in certain patients.

Whilst behavioural deficits were ameliorated by MPh administration to a level statistically indistinguishable from the HCs, few statistically significant differences in performance between TBI-MPh and TBI-placebo or HCs were shown. Ideally, more volunteers would increase the statistical power for behavioural and fMRI analyses, allowing for conformity to parametric testing to make more sound conclusions. It would be desirable to have a TBI cohort recovering from *severe* TBI only. A severely injured cohort may demonstrate greater behavioural deficits versus HCs and may demonstrate greater beneficial effects from MPh.

Considering the structural assumptions made about CSTC loops being the structural neurobiological underpinnings, future studies would utilise diffusion-based MRI to support activity and FC conclusions. Both anisotropic and diffusion metrics would provide further insight into potential structural connections potentially underlying neuromodulatory mechanisms contributing to executive function after TBI.

While the task order was randomised to avoid mental fatigue, a main consequence of TBI is chronic fatigue, which affects approximately 60% patients [197,198,199]. This cognitive challenge may have led to the heterogeneity in both the patients and HCs and made it difficult to reveal clearer statistically significant differences.

Here, only TBI patients received pharmacological intervention, as a single dose of MPh, and a placebo trial. MPh administration to HCs would allow for better experimental design and more evident behavioural and neurobiological improvements to compare to previous work. Neurofunctional differences could be compared between patients and controls, on and off MPh, thus providing further insight into the neuromodulation of regions contributing to executive function and planning after TBI and in healthy individuals.

## 5. Conclusions

This work demonstrates how TBI patients have deficiencies in executive function marked by visuospatial planning deficits in accuracy and reaction time versus HCs. It also shows how deficits are reflected in differences in activity and connectivity between both HCs and TBI-MPh versus TBI-placebo and how differences are ameliorated with MPh. It proposes that the striatum, thalamus, insula, and IFG are important for coordinated neurobiological function during an executive function task. MPh-driven enhancements in these regions are directed towards visuospatial planning and executive function in patients with chronic TBI. MPh may allow utilisation of areas that are less vulnerable to TBI, including the IFG and insulae, to enhance connectivity via subcortical structures to the frontoparietal network and the cerebellum to ameliorate executive dysfunction of chronic TBI. Acute MPh administration may provide such a dopaminergic neuro-modulatory mechanism via fronto-striatal and CSTC loops.

## Figures and Tables

**Table 1:** Patient information: time since injury (months), injury severity (Glasgow Coma Scale [GCS] at the scene), Glasgow Outcome Score Extended (GOSE), demographics, lesion information and acute CT scan results description for TBI patients.

Patient Number	Time since Injury (Months)	GSC Score	GSC Level	GOSE Score	Age at Scan	Sex	Scan Result (Acute)
2001	25	7	Severe	4	27	Male	Evidence of haemorrhage in both frontal lobes at gray/white matter interfaces and corpus callosum as well as the superior cerebellar cistern. No mass lesions.
2002	17	12	Moderate	3	55	Female	Subarachnoid haemorrhage in the sulci of the left frontoparietal convexity.
2003	14	5	Severe	4	29	Male	Multiple haemorrhagic contusions left temporal lobe. Haemorrhage left basal ganglia. Right thalamus. Right subcortical diffuse axonal injury.
2004	36	5	Severe	4	49	Female	Small, scattered petechial haemorrhages in both cerebral hemispheres. A slightly larger hemorrhage in the left temporal lobe (superior to the petrous ridge).
2006	32	7	Severe	4	19	Male	Subarachnoid haemorrhage in the left interpeduncular fossa and foramen magnum.
2007	9	14	Mild	5	58	Male	Haemorrhagic contusions in orbital frontal cortex. Subarachnoid blood in both cerebral convexities.
2008	10	3	Severe	3	23	Male	Subarachnoid haemorrhage in convexity sulci bilaterally and interpeduncular fossa.
2009	39	3	Severe	3	21	Male	N/A
2010	7	8	Severe	5	19	Male	R frontoparietal epidural haematoma. Small haemorrhagic contusions left inferior frontal, lateral orbitofrontal gyri and anterior aspect of left temporal lobe.
2011	27	8	Severe	4	49	Male	Haemorrhagic contusion left lentiform nucleus. Small focal lesion pons. Bilateral subcortical area frontal lobes. Signal change in corpus callosum.
2012	11	6	Severe	4	36	Male	Right temporal epidural haematoma, haemorrhagic contusions anterior aspect left temporal lobe, posterior inferior right frontal lobe. Scattered areas traumatic subarachnoid haemorrhage in interpeduncular fossa and some of the posterior convexity sulci of both hemispheres.
2013	25	7	Severe	4	26	Male	Intraventricular haemorrhage.
2015	41	N/A	N/A	5	34	Female	Right temporal/parietal contusion.
2016	7	10	Moderate	5	43	Male	Right subarachnoid haemorrhage and subdural haemorrhage.
2018	8	8	Severe	5	19	Female	Left temporal lobe contusion and left tentorial subdural haemorrhage.
2019	32	14	Mild	4	53	Male	Right subarachnoid haemorrhage and subdural haemorrhage. Haemorrhagic contusion right posterior temporal lobes. Multiple areas contusion superior frontal lobes and right cerebellar hemisphere, right temporal and inferior frontal lobes.

N/A: Not Available.

## Data Availability

The data supporting findings are available from the senior author upon reasonable request.

## Supplementary Materials

The following supporting information can be downloaded at: https://www.mdpi.com/article/10.3390/jcm13030771/s1, Reference [200] is cited in Supplementary Materials. Figure S1: Schematic representation (flowchart) of the protocols of the study. Figure S2: Neuropsychiatric differences between Healthy Controls (HCs), Patients with chronic TBI (TBI) all taken during their first visit and prior to receiving medication. Figure S3. CANTAB tests between Healthy Controls (HCs), Patients with chronic TBI (TBI) all taken during their first visit and prior to receiving medication. Figure S4: fMRI Tower of London task paradigm. Figure S5: Schematic representation of the potential communication loops and targets of Methylphenidate (MPh) during the Tower of London task. Figure S6: Twenty-three spherical Regions-of-interests and their coordinates used in task-modulated functional connectivity analyses. Figure S7: Significant differences in *3 moves* > *fixation* task-modulated functional connectivity (gPPI) in Healthy Controls vs patients with TBI Placebo for 4 regions-of-interest. Figure S8: Significant differences in *3 moves* > *fixation* task-modulated functional connectivity (gPPI) in TBI patients on Methylphenidate vs patients on Placebo & Healthy Controls. Table S1: Neuropsychiatric differences between Healthy Controls (HCs), Patients with chronic TBI all taken during their first visit and prior to receiving medication. Table S2: CANTAB tests between Healthy Controls (HCs), Patients with chronic TBI all taken during their first visit and prior to receiving medication. Table S3: Significant activation peaks for the *1 move* & *3 moves* versus *fixation* and count contrasts for each experimental group. Table S4: Significant activation peaks for the *1 move* & *3 moves* versus *fixation* contrasts for comparing each experimental group. Table S5: Significant activation peaks in *beyond*-network connectivity for the *3 moves* versus *fixation* contrasts while on-and-off Methylphenidate. Table S6: Significant activation peaks in *beyond*-network connectivity for the *3 moves* versus *fixation* contrasts for comparing the experimental groups. Supplementary Notes: Table S7: Pseudo-randomised order of attempted trials: *Count* (C), *1 move* (1), *2 moves* (2), *3 moves* (3), and *4 moves* (4) by Healthy Controls (HCs). Supplementary Notes: Table S8: Pseudo-randomised order of attempted trials: *Count* (C), *1 move* (1), *2 moves* (2), *3 moves* (3), and *4 moves* (4) by patients with chronic TBI on Methylphenidate (TBI-MPh). Supplementary Notes: Table S9: Pseudo-randomised order of attempted trials: *Count* (C), *1 move* (1), *2 moves* (2), *3 moves* (3), and *4 moves* (4) by patients with chronic TBI on placebo (TBI-placebo).
