# Supplementary material for: Methylphenidate Ameliorates Behavioural and Neurobiological Deficits in Executive Function for Patients with Chronic Traumatic Brain Injury
Source: J Clin Med. 2024 Jan 29;13(3):771. doi: 10.3390/jcm13030771 (PMC10856064; doi:10.3390/jcm13030771)
Supplement: Supplementary file 1 [file jcm-13-00771-s001.zip › jcm-2773130-supplementary.pdf]

Supplementary Material

Figure S1 – Schematic representation (flowchart) of the protocols of the study.

Figure S2 – Neuropsychiatric differences between Healthy Controls (HCs), Patients with chronic TBI (TBI) all taken during their first visit and prior to receiving medication.

Figure S3. CANTAB differences between Healthy Controls (HCs), Patients with chronic TBI (TBI) all taken during their first visit and prior to receiving medication.

Figure S4 – fMRI Tower of London task paradigm.

Figure S5 – Schematic representation of the potential communication loops and targets of Methylphenidate (MPh) during the Tower of London task.

Figure S6 – Twenty-three spherical Regions-of-interests and their coordinates used in task-modulated functional connectivity analyses.

Figure S7 – Significant differences in *3moves>fixation* task-modulated functional connectivity (gPPI) in Healthy Controls vs patients with TBI Placebo for 4 regions-of-interest.

Figure S8 – Significant differences in *3moves>fixation* task-modulated functional connectivity (gPPI) in TBI patients on Methylphenidate vs patients on Placebo & Healthy Controls.

Table S1 – Neuropsychiatric differences between Healthy Controls (HCs), Patients with chronic TBI all taken during their first visit and prior to receiving medication.

Table S2 – CANTAB differences between Healthy Controls (HCs), Patients with chronic TBI all taken during their first visit and prior to receiving medication.

Table S3 – Significant activation peaks for the *1move* & *3moves* versus *fixation* and *count* contrasts for each experimental group.

Table S4 – Significant activation peaks for the *1move* & *3moves* versus *fixation* contrasts for comparing each experimental group.

Table S5 – Significant activation peaks in *beyond*-network connectivity for the *3moves* versus *fixation* contrasts while on-and-off Methylphenidate.

Table S6 – Significant activation peaks in *beyond*-network connectivity for the *3moves* versus *fixation* contrasts for comparing the experimental groups.

Notes

Supplementary Notes: Table S7 - Pseudo-randomised order of attempted trials: *Count* (C), *1 move* (1), *2 moves* (2), *3 moves* (3), and *4 moves* (4) by Healthy Controls (HCs).

Supplementary Notes: Table S8 - Pseudo-randomised order of attempted trials: *Count* (C), *1 move* (1), *2 moves* (2), *3 moves* (3), and *4 moves* (4) by patients with chronic TBI on Methylphenidate (TBI-MPh).

Supplementary Notes: Table S9 - Pseudo-randomised order of attempted trials: *Count* (C), *1 move* (1), *2 moves* (2), *3 moves* (3), and *4 moves* (4) by patients with chronic TBI on placebo (TBI-placebo).

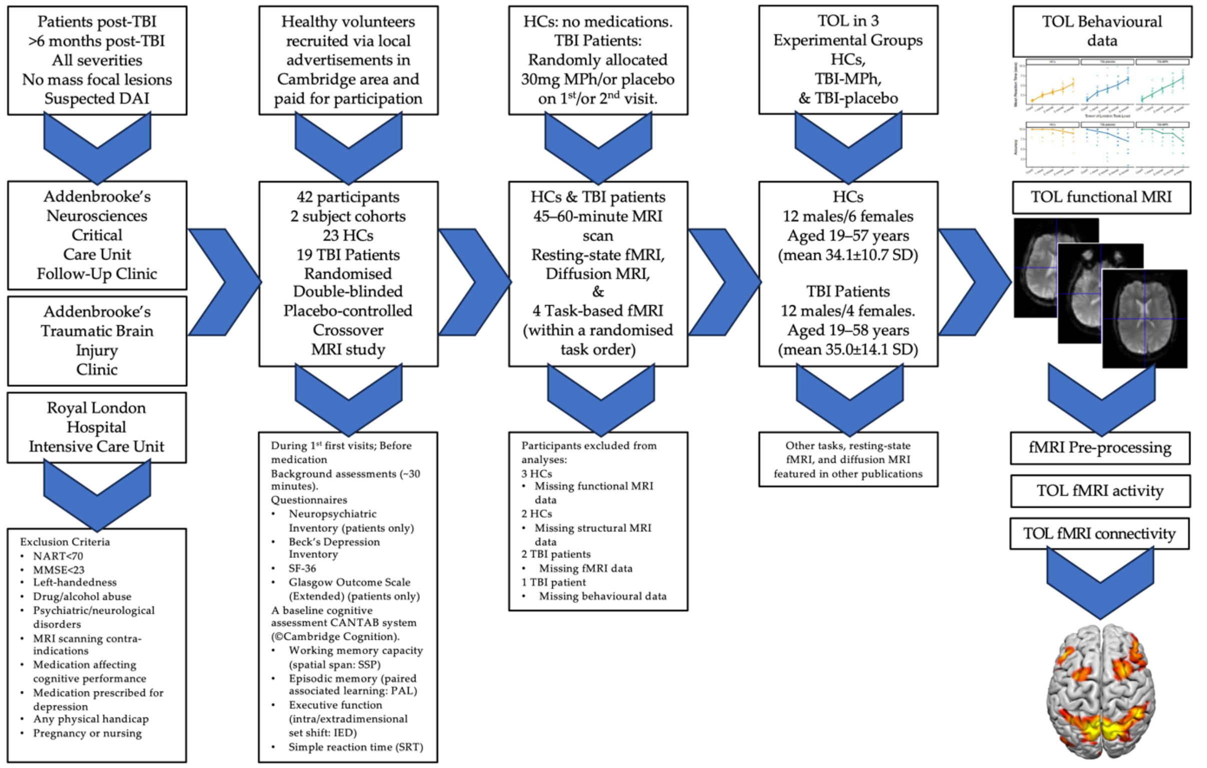

Figure S1. Schematic representation (flowchart) of the protocols of the study. Two experimental cohorts participated in a randomised, double-blinded, placebo-controlled, crossover Magnetic Resonance Imaging (MRI) study. Participants were referred from the Addenbrooke's Neurosciences Critical Care Unit Follow-Up Clinic, Addenbrooke's Traumatic Brain Injury Clinic, and Royal London Hospital Intensive Care Unit. The HC volunteers were recruited through local advertisements in the Cambridge area and paid for participation. Patients with a history of TBI (of all severities: *mild* through *moderate* to *severe*), without mass focal lesions on acute CT scans, not included in >3 research studies within a calendar year, were approached to voluntarily participate. Forty-two participants met the study criteria (23 Heathy Controls (HCs) and 19 patients). Both cohorts attended two sessions separated by 2-4 weeks. Only patients who were at least six-months post-TBI (i.e., chronic TBI), without mass focal lesions, and suspected DAI were included in this study. HCs received no pharmacological intervention. TBI patients were randomly allocated, via a Latin Square design, to receive one of two visually indistinguishable medications on their 1st visit with the other on their 2nd visit. These medications contained either 30mg Methylphenidate (MPh) or a placebo containing lactose. After a 75-minute delay to ensure peak plasma levels were reached, patients completed a 45–60-minute fMRI scan with both resting-state fMRI (rs-fMRI), diffusion MRI, and task-based fMRI, using a randomised task order. During their 1st visits and before receiving medication, background assessments, lasting ~30 minutes, were performed for both patients and HCs. These included questionnaires assessing current psychiatric status and quality of life (e.g., the Neuropsychiatric Inventory (patients only), Glasgow Outcome Scale Extended (patients only), Beck's Depression Inventory, SF-36). Additionally, a baseline cognitive assessment was conducted using the Cambridge Neuropsychological Test Automated Battery (CANTAB) system (©Cambridge Cognition) also before medication was administered. These tests assessed working memory capacity (spatial span: SSP), episodic memory (paired associated learning: PAL), executive function (intra/extradimensional set shift: IED), and simple reaction time (SRT). 3 HCs had missing fMRI data, and 2 missing structural MRI data. 2 patients had missing fMRI data, and 1 had missing behavioural data. Subsequently, these subjects were removed from further analyses. Thereafter, HCs consisted of 12 males/6 females. Patients consisted of 12 males/4 females. The fMRI-TOL-task used a randomised trial design consisting of five conditions inter-dispersed with fixation conditions. Trial stimulus onset time, reaction time, and response outcome during blocks were recorded for each participant. Simultaneous fMRI was recorded while participants attempted the task. This fMRI data was subsequently pre-processed, activity analysed, and functional connectivity calculated for further analyses.

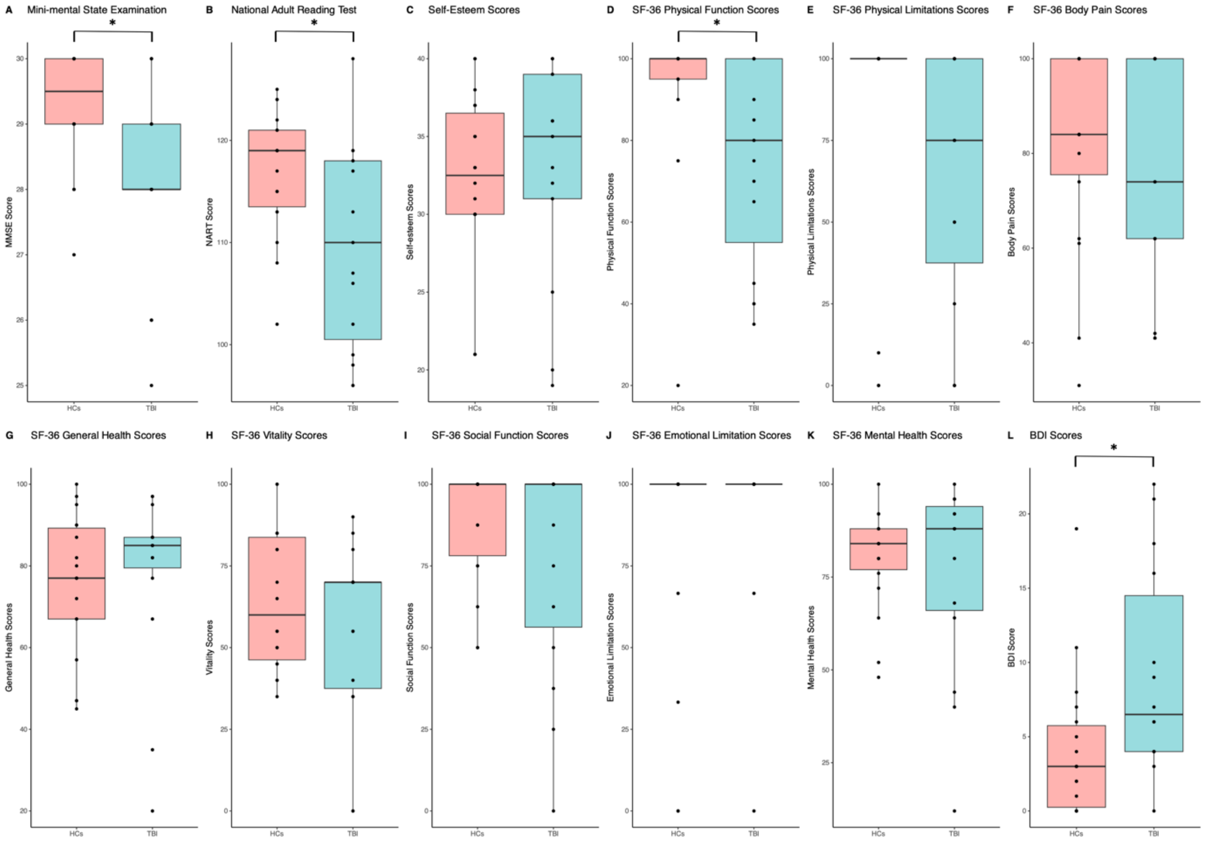

Figure S2. Neuropsychiatric differences between Healthy Controls (HCs), Patients with chronic TBI (TBI) all taken during their first visit and prior to receiving medication. **A** – Mini-mental state examination (MMSE) Scores of HCs and patients with chronic TBI. **B** – National Adult Reading Test (NART) Scores of HCs and patients with chronic TBI. **C** – Self-esteem Scores of HCs and patients with chronic TBI. **D** – Short Form 36 Health Survey Questionnaire (SF-36) Physical Function Scores of HCs and patients with chronic TBI. **E** – SF-36 Physical Limitations Scores of HCs and patients with chronic TBI. **F** – SF-36 Body Pain Scores of HCs and patients with chronic TBI. **G** – SF-36 General Health Scores of HCs and patients with chronic TBI. **H** – SF-36 Vitality Scores of HCs and patients with chronic TBI. **I** – SF-36 Social Function Scores of HCs and patients with chronic TBI. **J** – SF-36 Emotional Limitation Scores of HCs and patients with chronic TBI. **K** – SF-36 Mental Health Scores of HCs and patients with chronic TBI. **L** – Beck's Depression Inventory (BDI) Scores of HCs and patients with chronic TBI. | *ns* not significant | * Significant at $p < 0.05$. | ** Significant at $p < 0.005$. Results were analysed, calculated, and visualised using "ggstatsplot" (Patil, 2021).

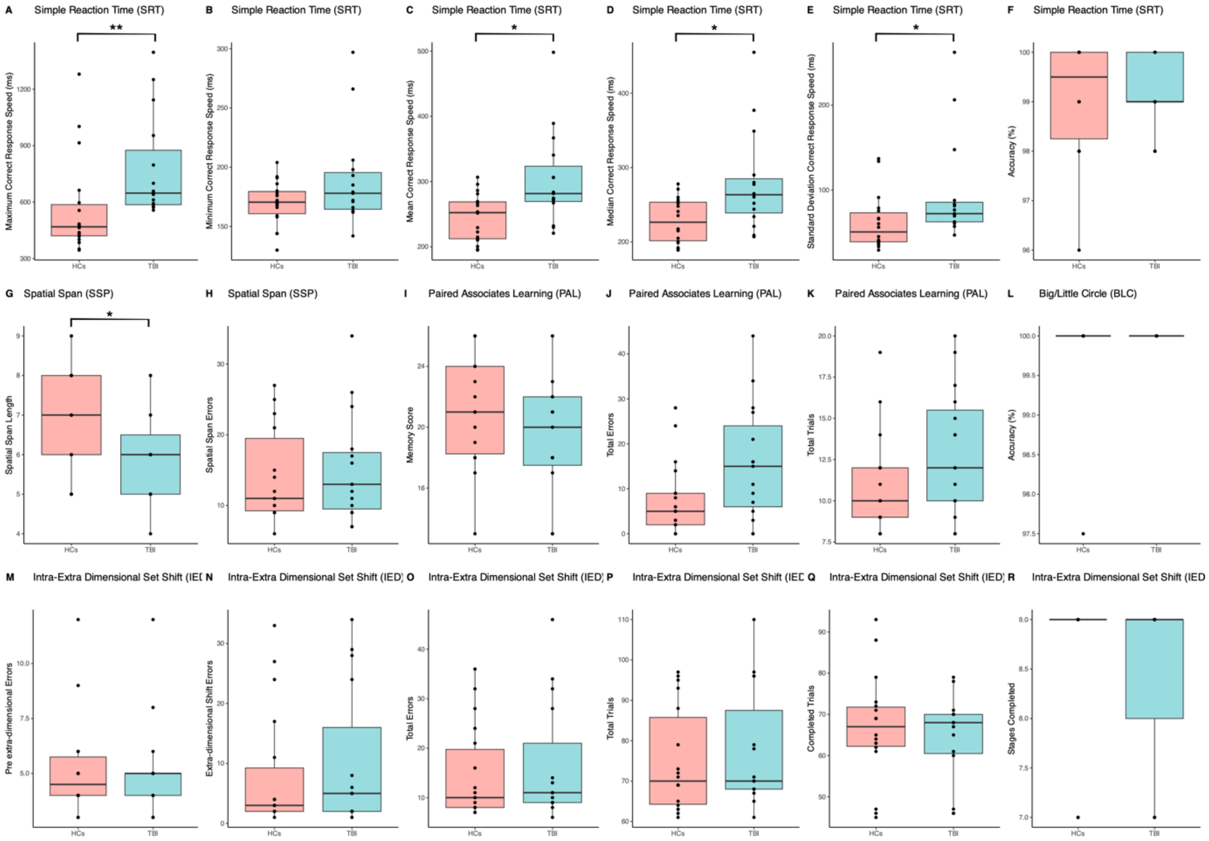

Figure S3. CANTAB tests between Healthy Controls (HCs), Patients with chronic TBI (TBI) all taken during their first visit and prior to receiving medication. Simple Reaction Time (SRT) i.e., **A** – maximum correct response speed, **B** – minimum correct response speed, ***C*** – mean correct response speed, **D** – median correct response speed, **E** – standard deviation correct response speed, and **F** – accuracy of HCs and patients with chronic TBI. Spatial Span (SSP) i.e., **G** – length, and **H** – errors of HCs and patients with chronic TBI. Paired Associates Learning (PAL) i.e., **I** – memory score, **J** – number of total errors, **K** – number of total trials of HCs and patients with chronic TBI. **L** – Big/Little Circle Accuracy of HCs and patients with chronic TBI. Intra-extra dimensional (IED) shift i.e., **M** – pre extra-dimensional errors, **N** – extra-dimensional shift errors, **O** – total errors, **P** – total trials, **Q** – completed trials, and **R** – completed stages of HCs and patients with chronic TBI. | *ns* not significant | * Significant at $p < 0.05$. | ** Significant at $p < 0.005$. Results were analysed, calculated, and visualised using "ggstatsplot" (Patil, 2021).

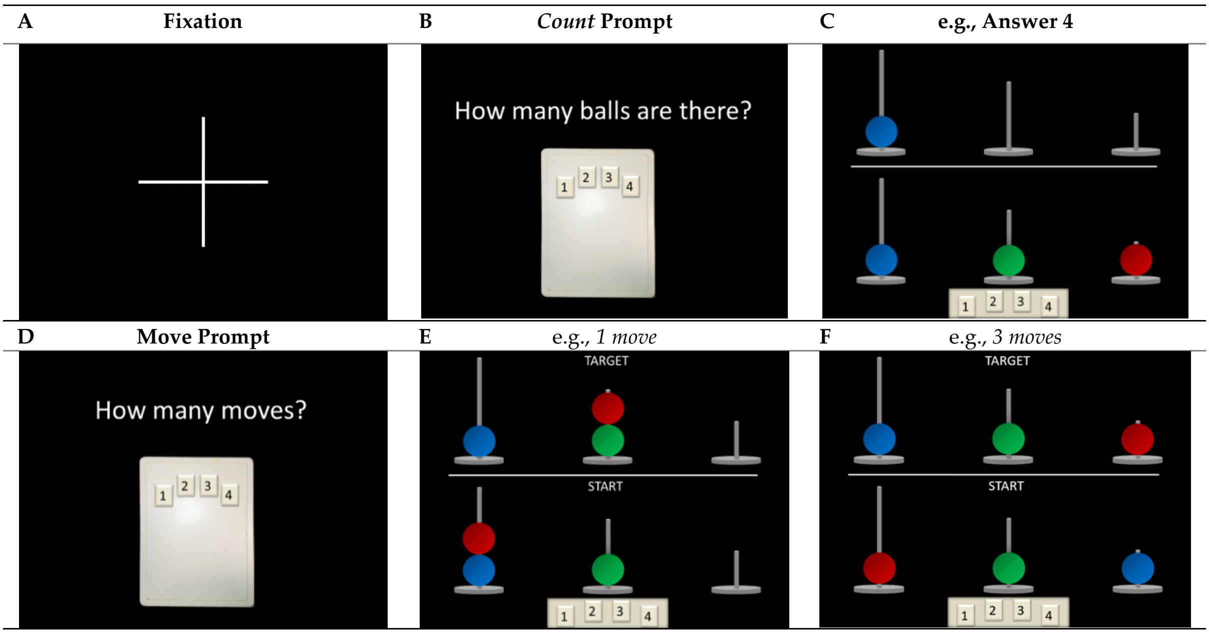

Figure S4. fMRI Tower of London task paradigm: **A** – fixation, **B** – Count Prompt, **C** – "*Count*" task example, **D** – Move Prompt, **E** – "*1 move*" example, **F** – "*3 moves*" example.

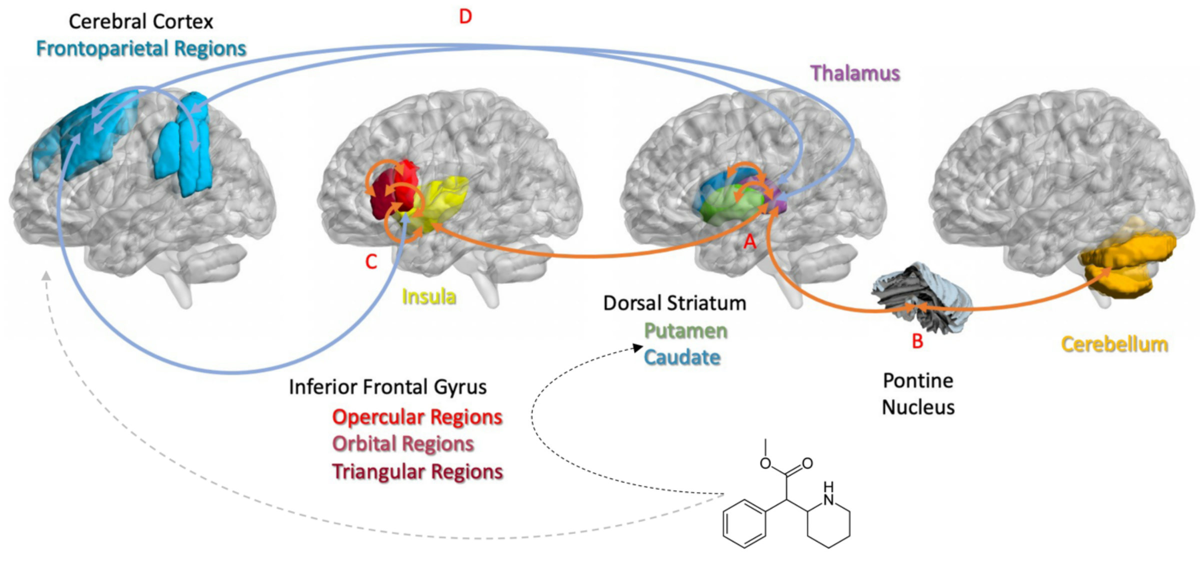

Figure S5. Schematic representation of the potential communication loops and targets of Methylphenidate (MPh) during the Tower of London task. Communication feedback loops (shown by orange arrows) between the dorsal striatum: putamen (green), caudate (blue) and the cerebellum shown on the left of this schematic representation. Methylphenidate primarily acts on regions of the basal ganglia and frontal cortex. **A** – Dorsal Striatal input from the putamen (green) and caudate nucleus (blue) is routed through the thalamus to various regions. **B** –This includes inputs to the subthalamic nucleus (STN) and via the Pontine Nucleus and onwards to the Cerebellar cortex (shown in amber). **C** – This also includes inputs towards the Insula (yellow) and onwards to the Inferior Frontal Gyrus (red) as these share strong reciprocal connections. **D** –These communication pathways (blue arrows) are in turn connected to regions of the frontoparietal network (turquoise) which has reciprocal feedback loops to the thalamus.

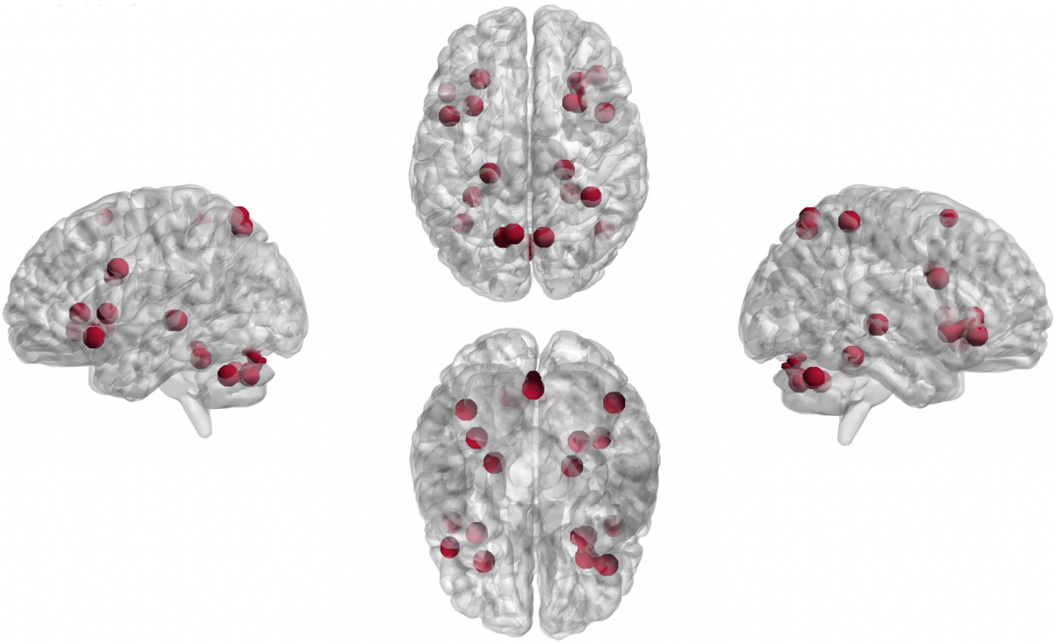

Area Name	Side	MNI Coordinates		
		X	Y	Z
Superior Frontal Gyrus	L	-20	8	64
	R	26	14	60
Precuneus	L	-8	-64	60
	R	10	-66	54
Superior Parietal Lobule	L	-14	-66	54
	R	38	-42	58
Opercular part of the Inferior Frontal Gyrus	L	-46	6	30
	R	46	8	26
Orbital part of the Inferior Frontal Gyrus	L	-48	20	-10
	R	42	30	-6
Insula	L	-28	28	4
	R	30	26	-2
Thalamus	L	-22	-28	0
	R	22	-26	0
Striatum (Putamen/Dorsal Striatum)	L	-32	12	4
	R	30	14	-4
Cerebellar Crus 1	L	-38	-58	-32
	R	46	-62	-30
Cerebellum Layer 4/5	L	-32	-42	-22
	R	26	-40	-18
Vermis 7		0	-72	-20
Vermis 8		0	-74	-22
Vermis 9		0	-70	-32

Figure S6. Twenty-three spherical Regions-of-interests and their coordinates used in task-modulated functional connectivity analyses.

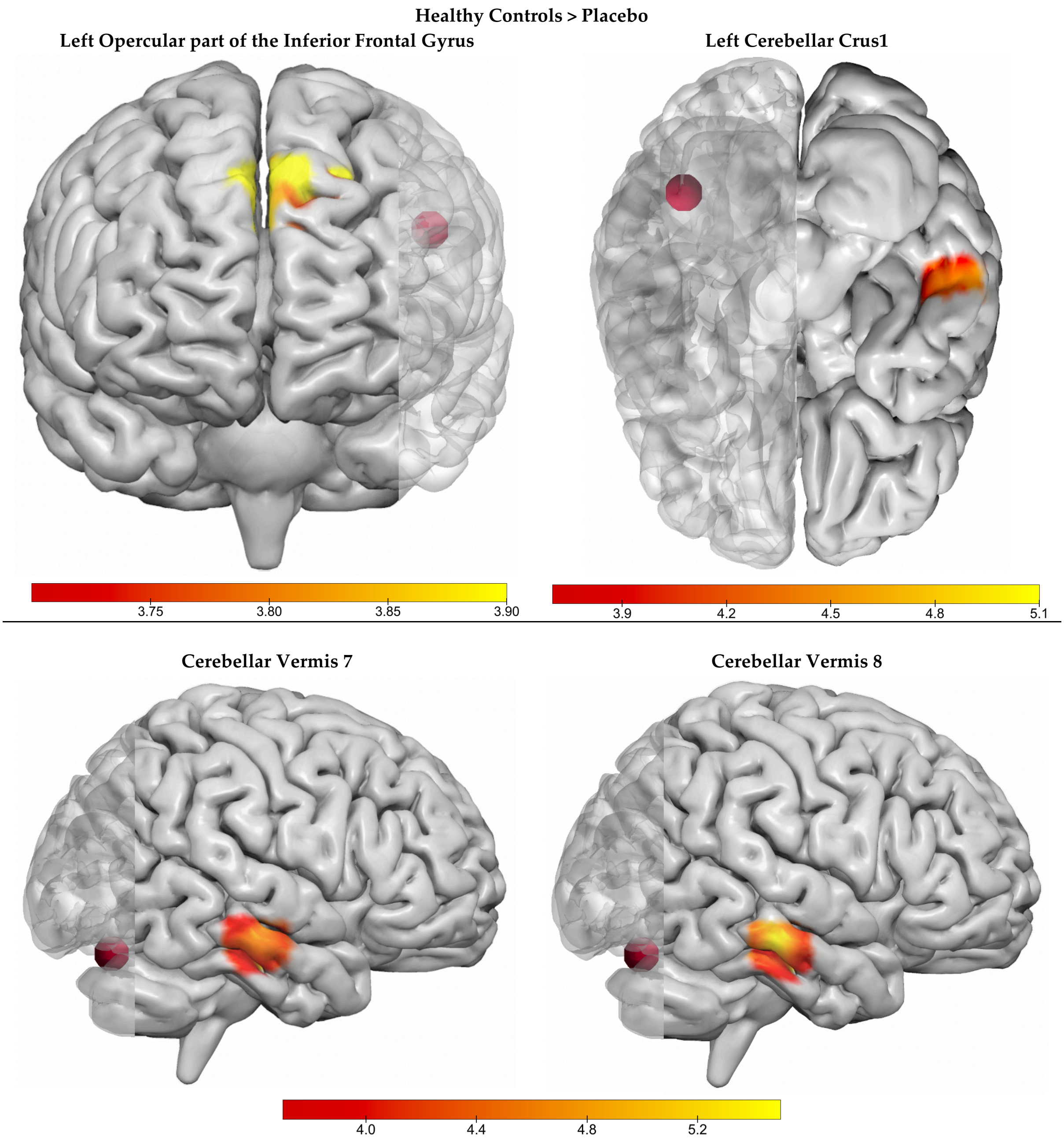

Figure S7. Significant differences in *3moves>fixation* task-modulated functional connectivity (gPPI) in Healthy Controls vs patients with TBI Placebo for 4 regions-of-interest (red spheres). Results are superimposed on a template supplied by Surf Ice (https://www.nitrc.org/projects/surfice/) and BrainNet Viewer (Xia *et al.*, 2013) (https://www.nitrc.org/projects/bnv/).

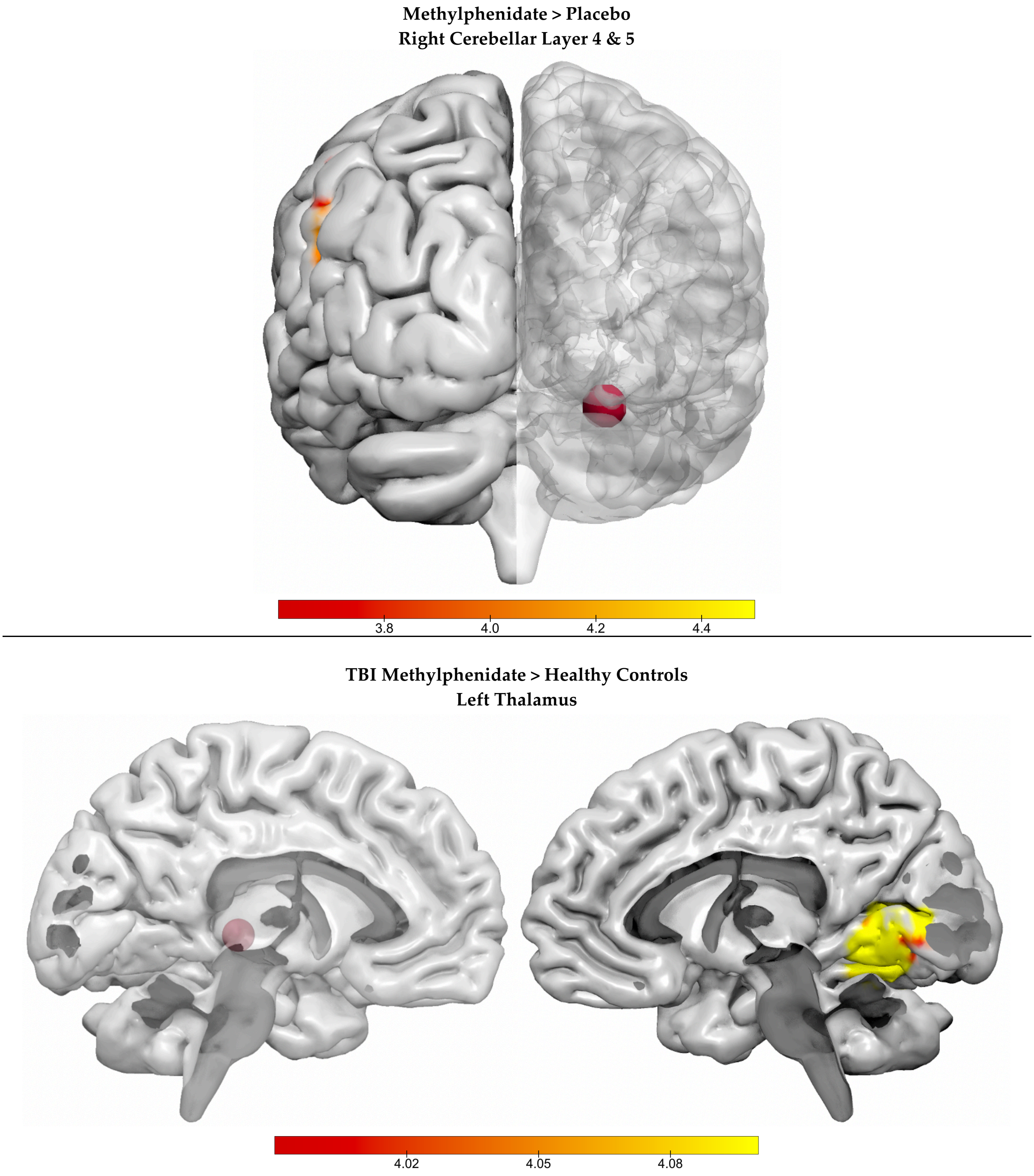

Figure S8. Significant differences in *3moves>fixation* task-modulated functional connectivity (gPPI) in in patients with TBI on Methylphenidate versus Placebo for Right Cerebellar Layer 4 & 5 (top) and patients on Methylphenidate versus Healthy Controls for Left Thalamus (both red spheres). Results are superimposed on a template supplied by Surf Ice (https://www.nitrc.org/projects/surfice/) and BrainNet Viewer (Xia *et al.*, 2013) (https://www.nitrc.org/projects/bnv/).

Table S1. Neuropsychiatric differences between Healthy Controls (HCs), Patients with chronic TBI all taken during their first visit and prior to receiving medication. Age of Subjects at Assessment, Mini-mental state examination (MMSE), National Adult Reading Test (NART), Short Form 36 Health Survey Questionnaire (SF-36) Scores for Physical Function, Physical Limitations, Body Pain, General Health, Vitality, Social Function, Emotional Limitations Mental Health as well as Beck's Depression Inventory (BDI) Scores between healthy controls and patients with chronic TBI. Results were analysed, calculated, and visualised using "ggstatsplot" (Patil, 2021).

Neuropsychiatric Domain	Corresponding Figure	$W_{Mann\text{-}Whitney}$	p	$r^{rank}_{biserial}$	$CI_{95\%}$	
MMSE	Fig. S2A	199.50	0.02	0.48	[0.12,	0.73]
NART	Fig. S2B	199.50	0.02	0.48	[0.12,	0.73]
Self-esteem	Fig. S2C	101.00	0.53	-0.14	[-0.51,	0.27]
SF-36 Physical Function	Fig. S2D	196.00	0.02	0.45	[0.09,	0.71]
SF-36 Physical Limitation	Fig. S2E	178.50	0.06	0.32	[-0.07,	0.63]
SF-36 Body Pain	Fig. S2F	144.00	0.75	0.07	[-0.32,	0.44]
SF-36 General Health	Fig. S2G	111.00	0.39	-0.18	[-0.52,	0.22]
SF-36 Vitality	Fig. S2H	156.00	0.46	0.16	[-0.24,	0.51]
SF-36 Social Function	Fig. S2I	156.50	0.40	0.16	[-0.24,	0.51]
SF-36 Emotional Limitations	Fig. S2J	122.50	0.52	-0.09	[-0.46,	0.30]
SF-36 Mental Health	Fig. S2K	121.00	0.62	-0.10	[-0.47,	0.29]
Beck's Depression Inventory	Fig. S2L	62.00	0.02	-0.51	[-0.75,	-0.15]

Table S2 – CANTAB differences between Healthy Controls (HCs), Patients with chronic TBI all taken during their first visit and prior to receiving medication. Simple Reaction Time (SRT) i.e., maximum correct response speed, minimum correct response speed, mean correct response speed, median correct response speed, standard deviation correct response speed, and accuracy of healthy controls and patients with chronic TBI. Spatial Span (SSP) i.e., length, and errors of healthy controls and patients with chronic TBI. Paired Associates Learning (PAL) i.e., memory score, number of total errors, number of total trials of healthy controls and patients with chronic TBI. Big/Little Circle Accuracy of healthy controls and patients with chronic TBI. Intra-extra dimensional (IED) shift i.e., pre extra-dimensional errors, extra-dimensional shift errors, total errors, total trials, completed trials, and completed stages of healthy controls and patients with chronic TBI. Results were analysed, calculated, and visualised using "ggstatsplot" (Patil, 2021).

CANTAB Domain	Corresponding Figure	$W_{Mann\text{-}Whitney}$	p	$r^{rank}_{biserial}$	$CI_{95\%}$	
SRT Maximum Correct Reaction Time	Fig. S3A	51.00	0.003	-0.62	[-0.81,	0.32]
SRT Minimum Correct Response Speed	Fig. S3B	101.00	0.23	-0.25	[-0.58,	0.14]
SRT Mean Correct Response Speed	Fig. S3C	57.00	0.005	-0.58	[-0.79,	-0.25]
SRT Median Correct Response Speed	Fig. S3D	61.50	0.008	-0.54	[-0.77,	-0.21]
SRT Standard Deviation Correct Response Speed	Fig. S3E	66.00	0.01	-0.51	[-0.75,	-0.16]
SRT Accuracy	Fig. S3F	138.00	0.92	0.02	[-0.36,	0.40]
SSP Length	Fig. S3G	193.00	0.03	0.43	[0.06,	0.70]
SSP Errors	Fig. S3H	125.00	0.73	-0.07	[-0.44,	0.32]
PAL Memory Score	Fig. S3I	167.50	0.24	0.24	[-0.15,	0.57]
PAL Total Errors	Fig. S3J	81.50	0.05	-0.40	[-0.68,	-0.02]
PAL Total Trials	Fig. S3K	88.00	0.09	-0.35	[-0.64,	0.04]
BLC Accuracy	Fig. S3L	127.50	0.39	-0.06	[-0.43,	0.33]
IED Pre extra-dimensional Errors	Fig. S3M	142.00	0.81	0.05	[-0.34,	0.42]
IED Extra-dimensional Shift Errors	Fig. S3N	117.00	0.52	-0.13	[-0.49,	0.26]
IED Total Errors	Fig. S3O	114.00	0.46	-0.16	[-0.51,	0.24]
IED Total Trials	Fig. S3P	119.50	0.59	-0.11	[-0.48,	0.28]
IED Completed Trials	Fig. S3Q	146.50	0.69	0.09	[-0.31,	0.45]
IED Completed Stages	Fig. S3R	148.50	0.51	0.10	[-0.29,	0.46]

Table S3 – Significant activation peaks for the *1move* & *3moves* versus *fixation* and count contrasts for each experimental group.

One-sample t-tests								
**Condition Contrast**	**Subject Group**	**Cluster Level**	**Cluster Extent**	**MNI Coordinates**			**t score**	**Peak location in Region**
		**p (FDR)**	**K**	**X**	**Y**	**Z**		
**1move > fixation**	**Healthy Controls**	0	8038	6	-80	18	7.84	Cuneus_R
				-36	-4	-18	7.17	Fusiform_L
				32	-52	-12	6.93	Fusiform_R
		0	859	50	-10	-16	5.45	Temporal_Mid_R
				50	-28	-4	5.21	Temporal_Mid_R
				48	0	-18	5.04	Temporal_Mid_R
		0.027	254	-8	8	56	5.14	Supp_Motor_Area_L
				-8	8	46	4.34	Supp_Motor_Area_L
				-10	16	34	3.94	Cingulum_Mid_L
		0.008	385	-52	-34	2	4.97	Temporal_Mid_L
				-54	-44	8	4.79	Temporal_Mid_L
		0.02	293	-42	-6	48	4.52	Precentral_L
				-54	-2	46	4.27	Precentral_L
				-50	-10	54	4.16	Postcentral_L
	**Patients on Methylphenidate**	0	16993	-12	-96	14	12.24	Occipital_Sup_L
				-20	-90	20	10.14	Occipital_Mid_L
				-32	-68	-16	10.13	Fusiform_L
		0	1959	-22	0	56	9.67	Frontal_Sup_L
				-44	0	32	7.45	Precentral_L
				-20	8	64	7.4	Frontal_Inf_Oper_L
		0	936	24	-6	52	7.53	n/a
				46	6	30	5.62	Precentral_R
				44	-2	40	5.45	Precentral_R
	**Patients on Placebo**	0	9241	-14	-94	14	13.43	Occipital_Sup_L
				-36	-80	-12	10.36	Fusiform_L
				32	-84	12	9.56	Occipital_Mid_R

		0	604	-44	2	32	7.98	Precentral_L
				-38	14	32	5.81	Frontal_Inf_Oper_L
				-28	-4	52	4.23	Frontal_Mid_L
		0.009	241	8	-26	-6	5.77	n/a
				-6	-28	-4	5.68	n/a
		0.011	214	26	-56	50	5.53	Parietal_Sup_R
**3 moves>fixation**	**Healthy Controls**	0	32806	32	-68	-10	12.16	Fusiform_R
				-16	-80	26	12.12	Occipital_Sup_L
				-26	-62	-6	11.95	Lingual_L
		0.009	368	0	-54	-34	7.06	Vermis_9
		0.009	372	2	-18	26	6.35	n/a
				-4	-34	26	4.49	Vermis_3
				8	-14	20	4.41	Thalamus_R
		0.001	644	-18	10	4	6.22	Putamen_L
				-32	18	10	4.59	Insula_L
				-30	26	2	4.45	Insula_L
		0.03	255	-8	-48	52	5.57	Precuneus_L
				8	-46	50	4.61	Precuneus_R
	**Patients on Methylphenidate**	0	20580	36	-86	14	14.99	Occipital_Mid_R
				-10	-98	18	14.16	Cuneus_L
				-26	-90	14	13.95	Occipital_Mid_L
		0	6847	-6	10	52	9.29	Supp_Motor_Area_L
				-46	2	34	9.19	Precentral_L
				24	-4	50	8.65	n/a
	**Patients on Placebo**	0	13218	-12	-96	14	11.86	Occipital_Sup_L
				32	-88	16	11.7	Occipital_Mid_R
				-20	-86	-10	9.94	Lingual_L
		0	2199	-48	2	30	8.07	Precentral_L
				-8	10	54	7.86	Supp_Motor_Area_L
				-30	-6	50	7.42	Precentral_L

		0	1100	-4	-22	2	7.24	Thalamus_L
				8	-28	-6	7.12	n/a
				-4	-12	2	6.58	Thalamus_L
		0	1116	40	-2	42	7.24	Precentral_R
				46	4	32	6.03	Precentral_R
				28	-4	50	5.82	Precentral_R

**One-sample t-tests**								
**Condition Contrast**	**Subject Group**	**Cluster Level**	**Cluster Extent**	**MNI Coordinates**			**t score**	**Peak location in Region**
		**p (FDR)**	**K**	**X**	**Y**	**Z**		
**1 move > count**	**Healthy Controls**	0	1792	10	-66	54	7.52	Precuneus_R
				-8	-64	60	7.47	Precuneus_L
				16	-66	60	7.41	Parietal_Sup_R
		0	907	46	-44	58	6.53	Parietal_Inf_R
				36	-40	32	6.27	n/a
				42	-42	48	5.97	Parietal_Inf_R
		0.011	334	-28	-26	30	6.43	n/a
				-22	-14	34	6.33	n/a
				-18	-2	40	6.14	n/a
		0.02	272	26	14	60	5.27	Frontal_Sup_R
				26	6	52	5.23	Frontal_Mid_R
	**Patients on Methylphenidate**	**NO SIGNFIICANT CLUSTERS**						

	Patients on Placebo	0.058	299	30	2	62	6.75	Frontal_Sup_R
				34	8	52	5.59	Frontal_Mid_R
		0.058	333	-40	-70	-28	5.39	Cerebelum_Crus1_L
				-30	-70	-26	5.1	Cerebelum_Crus1_L
				-36	-70	-46	3.98	Cerebelum_Crus2_L
		0.024	486	8	-64	56	5.06	Precuneus_R
				-2	-56	52	4.84	Precuneus_L
				-14	-70	56	4.66	Parietal_Sup_L
**3 moves > count**	**Healthy Controls**	**0**	**5175**	**14**	**-64**	**52**	**9.61**	Parietal_Sup_R
				-10	-66	54	9.03	Precuneus_L
				32	-38	32	8.29	n/a
		0	667	26	8	54	8.15	Frontal_Mid_R
		0.005	369	-26	-4	36	7.75	n/a
				-20	-10	32	6.02	n/a
				-20	-2	42	5.56	n/a
		0.004	408	-40	-68	-30	6.64	Cerebelum_Crus1_L
				-28	-62	-32	6.27	Cerebelum_Crus1_L
				-20	-64	-32	5.35	Cerebelum_Crus1_L
		0.008	322	-30	4	58	6.21	Frontal_Mid_L
				-20	8	64	5.81	Frontal_Sup_L
				-18	0	56	4.51	Frontal_Sup_L
	**Patients on Methylphenidate**	0	1592	-8	-60	52	9.15	Precuneus_L
				6	-62	52	8.93	Precuneus_R
				-14	-66	54	7.61	Parietal_Sup_L
		0	682	38	-42	58	8.37	Parietal_Sup_R
				58	-34	46	5.39	SupraMarginal_R
		0.015	318	26	2	62	7.9	Frontal_Sup_R

				24	10	58	6.49	Frontal_Sup_R
	Patients on Placebo	0	916	28	0	62	7.77	Frontal_Sup_R
				40	26	34	6.84	Frontal_Mid_R
				36	8	52	5.58	Frontal_Mid_R
		0	734	36	-40	48	7.72	Parietal_Inf_R
				40	-50	56	7.23	Parietal_Inf_R
		0	1732	2	-66	52	7.6	Precuneus_R
				-10	-62	60	7.35	Precuneus_L
				-8	-56	54	6.84	Precuneus_L
		0	2868	46	-62	-30	6.94	Cerebelum_Crus1_R
				-38	-58	-32	6.57	Cerebelum_Crus1_L
				-26	-66	-28	6.39	Cerebelum_6_L
		0.018	324	-24	0	66	6.73	Frontal_Sup_L
				-24	8	60	6.58	Frontal_Mid_L

Table S4 – Significant activation peaks for the *1move* & *3moves* versus *fixation* contrasts for comparing each experimental group.

Group Comparison	Condition Contrast	Cluster Level	Cluster Extent	MNI Coordinates			t score	Peak location in Region
**Two-Sample t-tests**								
		p (FDR)	K	X	Y	Z		
Healthy Controls > Patients on Placebo	1move>fixation	0.091	415	-50	18	-12	5.75	Temporal_Pole_Sup_L
				-54	10	-12	4.74	Temporal_Pole_Sup_L
				-38	22	-20	4.59	Frontal_Inf_Orb_L
		0.119	322	42	30	-6	5.21	Frontal_Inf_Orb_R
				38	20	-24	4.49	Temporal_Pole_Sup_R
	3moves>fixation	0.004	751	4	-84	22	6.34	Cuneus_R
				12	-80	30	5.38	Calcarine_R
				-14	-80	26	4.86	Occipital_Sup_L
Healthy Controls > Patients on Methylphenidate	1move>fixation	NO SIGNFICANT CLUSTERS						
	3moves>fixation	NO SIGNFICANT CLUSTERS						
**Paired-sample t-tests**								
Patients on Methylphenidate > Patients on Placebo	1move>fixation	0.025	339	42	20	-8	5.01	Insula_R
				38	16	-2	4.72	Insula_R
				48	20	-2	4.67	Frontal_Inf_Oper_R
		0.02	392	8	-84	20	4.82	Cuneus_R
				16	-80	18	4.45	Calcarine_R
				-6	-92	16	4.28	Cuneus_L
		0.057	236	-48	16	6	4.78	Frontal_Inf_Oper_L
				-38	8	4	4.58	Insula_L
				-32	18	8	4.27	Insula_L

		0.02	395	20	-46	-2	4.7	Lingual_R
				14	-38	-6	4.4	Parahippocampal_R
				24	-50	-8	4.19	Lingual_R
		0.057	252	-12	-58	0	4.69	Lingual_L
				-4	-62	4	4.27	Vermis_4_5
				-10	-72	2	4.06	Lingual_L
		0.103	243	8	-86	22	5.96	Cuneus_R
	**3moves>fixation**			18	-76	14	4.87	Calcarine_R
				32	-72	16	4.7	Occipital_Mid_R

Table S5 – Statistically significant activation peaks in *beyond*-network connectivity for the *3moves* versus *fixation* contrasts while on-and-off Methylphenidate.

**Paired-Sample t-tests – Psychophysiological Interaction – 3 moves>fixation**								
**Group Comparison**	**Region-of-Interest (Height Threshold)**	**Cluster Level**	**Cluster Extent**		**MNI Coordinates**		**t score**	**Peak location in Region**
		**p (FDR)**	**K**	**X**	**Y**	**Z**		
**Patients on Methylphenidate > Patients on Placebo**	**Left Superior Parietal Lobule (p = 0.001)**	0.003	624	54	-34	42	6.04	SupraMarginal_R
				36	-42	48	5.47	Parietal_Inf_R
				38	-32	44	4.79	SupraMarginal_R
	**Left Precuneus (p = 0.005)**	0.062	736	54	-28	36	5.99	SupraMarginal_R
				44	-26	42	4.8	Postcentral_R
				56	-38	46	4.8	SupraMarginal_R
	**Left Thalamus (p = 0.005)**	0.005	1134	40	-84	20	5.14	Occipital_Mid_R
				26	-88	30	4.53	Occipital_Sup_R
				18	-100	4	3.98	Calcarine_R
		0.002	1467	-16	-84	-2	4.97	Lingual_R
				-8	-92	0	4.28	Calcarine_L

				26	-62	-2	4.07	Lingual_R
	**Right Thalamus (p = 0.005)**	0.05	668	-10	-52	-44	6.19	Cerebellum_9_L
				-16	-76	-24	4.85	Cerebellum_Crus1_L
				-20	-68	-22	4.66	Cerebellum_6_L
	**Right Cerebellar Crus1 (p = 0.005)**	0.014	831	-12	-76	-18	5.1	Cerebellum_6_L
				2	-70	-28	4.82	Vermis_7
				-8	-70	-10	4.49	Cerebellum_6_L
	**Cerebellar Vermis 7 (p = 0.001)**	0.035	857	-22	-94	22	4.02	Occipital_Sup_L
				-10	-96	26	3.88	Cuneus_L
				-36	-80	4	3.87	Occipital_Mid_L
	**Cerebellar Vermis 8 (p = 0.005)**	0	1937	-8	-82	6	5.09	Calcarine_L
				-22	-94	22	4.63	Occipital_Sup_L
				-10	-96	26	4.43	Cuneus_L

Table S6 – Statistically significant activation peaks in *beyond*-network connectivity for the *3moves* versus *fixation* contrasts for comparing the experimental groups.

Two- and Paired-Sample t-tests – generalised Psychophysiological Interaction – 3 moves > fixation

Group Comparison	Region-of-Interest (Height Threshold)	Cluster Level	Cluster Extent	MNI Coordinates			t score	Peak location in Region
		p (FDR)	K	X	Y	Z		
Healthy Controls > Patients on Placebo	Left Opercular part of the Inferior Frontal Gyrus (p = 0.001)	0.01	76	2	-20	18	7.56	n/a
		0.01	68	-4	46	42	7.06	Frontal_Sup_Medial_L
				4	50	34	4.48	Frontal_Sup_Medial_R
		0.01	64	-14	36	40	5.45	Frontal_Sup_L
				-16	28	38	5.28	n/a
	Left Cerebellar Crus1 (p = 0.001)	0.011	79	58	-26	-20	7.34	Temporal_Inf_R
				52	-26	-26	4.95	Temporal_Inf_R
	Cerebellar Vermis 7 (p = 0.001)	0.008	77	68	-16	-16	6.29	Temporal_Mid_R
				58	-22	-20	5.17	Temporal_Inf_R
	Cerebellar Vermis 8 (p = 0.001)	0.007	79	68	-24	-16	8.41	Temporal_Mid_R
				58	-22	-20	4.93	Temporal_Inf_R
Patients on Methylphenidate > Healthy Controls	Left Thalamus (p = 0.001)	0	123	18	-64	4	8.71	Lingual_R
				18	-56	-6	6.34	Frontal_Sup_Orb_R
				20	-62	-12	5.22	Fusiform_R
Patients on Methylphenidate > Patients on Placebo	Right Cerebellar Layers 4&5 (p = 0.001)	0.044	66	-48	-56	32	5.13	Angular_L
				-44	-50	46	4.42	Parietal_Inf_L

Supplementary Notes: TOL task stimulus presentation

Tower of London (TOL) involves moving coloured balls within a limited number of moves in order to achieve a target configuration. (e.g. Shallice, 1982). In this implementation of the task volunteers were presented with two configurations of 3 coloured balls (blue, red, green) in 3 pegs. The first peg can hold a maximum of three balls, the second peg, two and the third peg, one. The task screen was preceded by a screen providing instructions in the form of a question. For the counting task volunteers were asked how many balls are present on the screen. For the TOL task participants were asked how many moves it takes to go from the lower configuration (START) to the top (TARGET).

Prior to the scanning session, one of each trial type were presented to volunteers to familiarise them with the experiment. The scanning session follows an event related design whereby counting and moving TOL trials were pseudo-randomised so the same trial did not appear more than two consecutive times. Counting trials last for 7500ms with 2500ms for the instruction and 5000ms for the counting. Moving TOL trials lasted for 17500 ms. A screen with the instruction was presented for 2500 ms and then the TOL or counting task appeared for 15000ms maximum. If participants did not respond within this time the next trial appeared on the screen and this trial was marked as missed-incorrect response.

There were 10 counting, 10 1-move, 10 2-moves, 10 3-moves, and 10 4-moves trials giving a total of 50 trials. These were interspersed with fixation trials. Between both TOL trials and counting trials after the participant responded the screen was replaced with a fixation cross. The participants were instructed before the trial to wait until the next trials started if they saw a fixation cross. There was an "End of task" visual display at the end of the task. Taken together, the total experiment duration when including forty moving-TOL trials and ten counting trials was ((40x17500=700000ms) + (10x7500=75000) = 775000ms) = 12.916 minutes = ~12 minutes 55 seconds."

Supplementary Notes: Table S7 - Pseudo-randomised order of attempted trials: *Count* (C), *1 move* (1), *2 moves* (2), *3 moves* (3), and *4 moves* (4) by Healthy Controls (HCs).

**Control Number**	**1001**	**1003**	**1004**	**1005**	**1006**	**1007**	**1008**	**1010**	**1011**	**1012**	**1013**	**1015**	**1016**	**1019**	**1020**	**1021**	**1022**	**1023**
**MPh Visit**	n/a	n/a	n/a	n/a	n/a	n/a	n/a	n/a	n/a	n/a	n/a	n/a	n/a	n/a	n/a	n/a	n/a	n/a
**Trial 1**	1	2	2	1	3	2	4	3	C	3	1	2	3	4	1	4	C	2
**Trial 2**	4	3	C	C	2	4	3	C	4	2	4	1	C	3	3	1	1	4
**Trial 3**	3	1	4	3	1	3	1	4	3	1	C	C	1	1	4	C	2	1
**Trial 4**	2	4	3	4	4	1	2	1	2	C	3	3	2	2	2	3	4	C
**Trial 5**	C	C	1	2	C	C	C	2	1	4	2	4	4	C	C	2	3	3
**Trial 6**	4	2	4	C	C	1	4	3	C	3	3	1	3	4	3	4	4	2
**Trial 7**	2	1	1	4	2	3	1	1	3	C	C	2	C	3	C	3	C	4
**Trial 8**	3	4	3	2	3	2	3	2	2	1	2	3	4	1	2	2	3	1
**Trial 9**	C	C	2	3	4	4	C	4	4	4	1	4	1	2	4	1	1	3
**Trial 10**	1	3	C	1	1	C	2	C	1	2	4	C	2	C	1	C	2	C
**Trial 11**	2	1	C	C	C	4	2	C	C	C	2	2	3	4	1	4	4	2
**Trial 12**	C	3	2	3	2	1	4	2	2	3	4	3	C	C	3	1	3	1
**Trial 13**	1	4	3	2	1	3	C	3	1	1	3	C	1	3	2	2	1	3
**Trial 14**	3	C	4	1	4	2	1	4	4	4	1	4	4	1	C	C	2	4
**Trial 15**	4	2	1	4	3	C	3	1	3	2	C	1	2	2	4	3	C	C
**Trial 16**	1	1	3	3	3	1	2	1	C	4	3	2	3	4	4	4	C	2
**Trial 17**	2	3	1	C	4	4	1	C	4	2	2	1	C	1	3	1	2	C
**Trial 18**	C	2	C	4	C	2	3	3	2	C	1	C	1	C	2	2	3	4
**Trial 19**	3	4	2	1	1	C	C	2	3	1	C	3	2	3	C	3	1	3
**Trial 20**	4	C	4	2	2	3	4	4	1	3	4	4	4	2	1	C	4	1
**Trial 21**	4	4	4	C	2	4	C	1	4	1	1	3	4	3	3	1	4	1
**Trial 22**	C	C	3	3	3	C	1	C	2	C	2	4	2	C	C	C	3	3
**Trial 23**	2	2	C	1	C	3	4	4	3	2	C	C	3	4	2	3	C	4
**Trial 24**	3	1	2	4	1	1	3	2	1	4	4	1	C	2	1	2	2	2
**Trial 25**	1	3	1	2	4	2	2	3	C	3	3	2	1	1	4	4	1	C
**Trial 26**	3	3	4	3	4	3	1	3	4	2	4	3	4	4	3	2	4	2
**Trial 27**	4	C	1	1	1	C	3	C	2	1	1	4	2	C	4	4	3	1
**Trial 28**	1	1	2	C	3	1	2	4	C	C	2	C	3	3	1	3	1	3
**Trial 29**	2	4	C	4	C	2	4	2	1	4	3	1	C	1	C	C	2	4
**Trial 30**	C	2	3	2	2	4	C	1	3	3	C	2	1	2	2	1	C	C
**Trial 31**	2	1	2	2	4	C	C	C	2	3	4	2	4	1	2	4	2	4
**Trial 32**	3	2	C	C	1	2	3	2	4	2	3	1	1	3	C	C	C	1
**Trial 33**	4	C	4	4	2	3	4	1	1	1	2	C	3	C	4	2	3	C
**Trial 34**	C	4	3	3	C	1	2	3	C	4	1	4	2	2	3	1	1	3
**Trial 35**	1	3	1	1	3	4	1	4	3	C	C	3	C	4	1	3	4	2
**Trial 36**	3	3	1	2	4	4	2	1	1	1	2	C	2	1	3	3	1	3
**Trial 37**	C	C	4	4	C	3	C	2	4	3	C	4	3	3	C	C	C	4
**Trial 38**	4	1	2	1	1	2	1	4	C	4	4	3	4	2	1	4	4	1
**Trial 39**	1	2	3	3	2	C	3	3	3	C	3	1	C	C	4	2	2	C
**Trial 40**	2	4	C	C	3	1	4	C	2	2	1	2	1	4	2	1	3	2
**Trial 41**	1	1	3	1	2	C	4	1	4	4	2	1	3	4	2	C	4	2
**Trial 42**	4	2	C	C	4	4	3	4	C	2	3	3	2	1	3	4	2	C
**Trial 43**	3	C	2	3	3	3	C	C	2	C	C	4	C	3	C	3	1	3
**Trial 44**	2	4	4	2	1	1	2	3	1	3	4	C	1	2	4	2	3	1
**Trial 45**	C	3	1	4	C	2	1	2	3	1	1	2	4	C	1	1	C	4
**Trial 46**	2	C	3	2	C	1	3	4	1	2	C	1	3	C	3	2	C	3
**Trial 47**	C	3	2	C	2	3	C	1	4	4	1	C	4	3	2	1	1	4
**Trial 48**	4	1	1	1	4	4	4	C	C	C	4	2	1	2	4	C	2	1
**Trial 49**	3	2	4	3	3	C	2	3	2	3	3	4	2	4	C	3	4	2
**Trial 50**	1	4	C	4	1	2	1	2	3	1	2	3	C	1	1	4	3	C

Supplementary Notes: Table S8 - Pseudo-randomised order of attempted trials: *Count* (C), *1 move* (1), *2 moves* (2), *3 moves* (3), and *4 moves* (4) by patients with chronic TBI on Methylphenidate (TBI-MPh).

**Patient Number**	**2001**	**2002**	**2003**	**2004**	**2006**	**2007**	**2008**	**2009**	**2010**	**2011**	**2012**	**2013**	**2015**	**2016**	**2018**	**2019**
**MPh Visit**	1	1	2	2	1	1	2	1	1	1	2	2	1	2	1	1
**Trial 1**	3	1	C	3	3	4	C	2	3	2	4	2	3	C	1	4
**Trial 2**	2	C	2	4	4	1	4	C	4	1	2	3	4	4	2	1
**Trial 3**	1	2	4	C	2	3	2	4	C	4	C	C	1	1	3	3
**Trial 4**	C	3	1	1	1	2	3	3	1	C	1	4	C	3	4	C
**Trial 5**	4	4	3	2	C	C	1	1	2	3	3	1	2	2	C	2
**Trial 6**	1	4	1	1	C	4	C	2	1	1	4	C	2	C	C	3
**Trial 7**	3	3	3	3	1	3	3	1	2	C	1	1	4	1	1	1
**Trial 8**	4	C	C	4	4	1	4	C	3	4	3	3	1	3	3	2
**Trial 9**	2	1	2	2	3	2	2	3	4	2	C	4	3	2	2	4
**Trial 10**	C	2	4	C	2	C	1	4	C	3	2	2	C	4	4	C
**Trial 11**	4	2	1	2	1	4	C	C	4	2	2	2	3	C	1	4
**Trial 12**	2	1	2	C	C	C	2	2	C	3	3	C	4	1	3	2
**Trial 13**	C	4	C	4	4	2	3	1	2	4	1	4	1	2	C	C
**Trial 14**	3	3	4	3	2	1	4	4	3	C	4	3	2	3	2	1
**Trial 15**	1	C	3	1	3	3	1	3	1	1	C	1	C	4	4	3
**Trial 16**	3	1	2	3	1	4	C	C	C	4	2	3	3	C	1	4
**Trial 17**	4	4	3	4	3	1	4	3	3	3	C	C	4	4	2	1
**Trial 18**	C	2	C	C	C	2	2	2	1	1	4	1	C	2	3	2
**Trial 19**	2	3	4	2	2	C	1	1	2	C	1	4	1	3	C	3
**Trial 20**	1	C	1	1	4	3	3	4	4	2	3	2	2	1	4	C
**Trial 21**	1	4	2	1	4	3	4	4	1	1	C	C	3	1	C	3
**Trial 22**	3	2	4	2	2	2	2	2	2	3	4	1	C	2	4	2
**Trial 23**	C	1	C	3	1	1	1	3	3	C	1	4	1	C	3	1
**Trial 24**	4	3	1	C	3	C	3	1	C	2	2	2	2	4	1	4
**Trial 25**	2	C	3	4	C	4	C	C	4	4	3	3	4	3	2	C
**Trial 26**	C	3	1	4	4	4	C	4	4	2	1	4	4	1	C	3
**Trial 27**	1	C	2	C	1	C	1	C	1	4	3	2	C	2	3	C
**Trial 28**	4	2	C	2	C	2	3	3	2	1	4	C	3	C	1	2
**Trial 29**	2	1	3	3	2	1	4	1	3	C	2	3	2	4	2	4
**Trial 30**	3	4	4	1	3	3	2	2	C	3	C	1	1	3	4	1
**Trial 31**	4	2	4	2	3	1	2	3	2	4	C	4	3	C	3	1
**Trial 32**	1	4	1	3	1	4	4	C	3	1	2	C	1	4	2	4
**Trial 33**	2	3	3	C	2	C	C	4	1	C	1	2	C	2	4	2
**Trial 34**	3	1	2	1	4	3	3	1	C	3	4	1	4	1	1	C
**Trial 35**	C	C	C	4	C	2	1	2	4	2	3	3	2	3	C	3
**Trial 36**	C	3	C	2	2	1	2	C	C	1	2	2	1	3	2	C
**Trial 37**	2	2	3	3	4	3	3	2	1	4	4	4	2	1	C	3
**Trial 38**	1	1	4	C	C	C	1	1	3	3	3	1	4	2	1	1
**Trial 39**	3	C	2	4	1	2	C	3	4	C	C	3	C	4	4	2
**Trial 40**	4	4	1	1	3	4	4	4	2	2	1	C	3	C	3	4
**Trial 41**	2	1	C	C	1	4	C	2	C	3	3	3	2	C	C	3
**Trial 42**	1	C	1	4	4	1	1	C	1	2	2	1	4	1	2	C
**Trial 43**	4	2	2	3	3	C	2	1	3	C	1	4	C	3	4	2
**Trial 44**	C	3	3	2	C	2	4	4	4	1	C	2	3	4	3	1
**Trial 45**	3	4	4	1	2	3	3	3	2	4	4	C	1	2	1	4
**Trial 46**	4	2	2	4	3	C	1	1	3	4	3	C	2	4	2	C
**Trial 47**	2	1	4	3	C	4	4	3	2	C	4	4	3	3	1	4
**Trial 48**	C	3	3	2	4	3	3	4	4	3	C	3	1	C	C	1
**Trial 49**	3	C	1	C	1	2	2	C	C	1	1	1	4	2	3	2
**Trial 50**	1	4	C	1	2	1	C	2	1	2	2	2	C	1	4	3

Supplementary Notes: Table S9 - Pseudo-randomised order of attempted trials: *Count* (C), *1 move* (1), *2 moves* (2), *3 moves* (3), and *4 moves* (4) by patients with chronic TBI on placebo (TBI-placebo).

**Patient Number**	**2001**	**2002**	**2003**	**2004**	**2006**	**2007**	**2008**	**2009**	**2010**	**2011**	**2012**	**2013**	**2015**	**2016**	**2018**	**2019**
**Placebo Visit**	2	2	1	1	2	2	1	2	2	2	1	1	2	1	2	2
**Trial 1**	4	4	1	4	1	4	1	4	C	2	1	3	C	4	1	4
**Trial 2**	2	3	C	3	C	1	2	2	2	C	C	C	4	3	4	3
**Trial 3**	3	1	4	C	4	2	C	3	4	1	2	4	3	1	2	C
**Trial 4**	1	2	2	1	2	3	3	1	3	4	4	1	1	C	C	1
**Trial 5**	C	C	3	2	3	C	4	C	1	3	3	2	2	2	3	2
**Trial 6**	2	2	3	C	4	4	1	4	1	3	1	3	4	3	C	4
**Trial 7**	4	4	1	2	1	3	4	1	4	C	C	C	1	1	4	3
**Trial 8**	1	1	C	3	3	C	3	2	3	2	3	4	2	C	2	C
**Trial 9**	C	C	4	4	2	2	2	C	C	1	2	2	3	2	3	1
**Trial 10**	3	3	2	1	C	1	C	3	2	4	4	1	C	4	1	2
**Trial 11**	C	C	3	C	1	4	4	2	2	3	1	C	C	4	1	4
**Trial 12**	3	3	C	4	3	C	3	4	C	1	2	3	1	3	2	C
**Trial 13**	1	2	1	2	2	3	C	1	4	2	3	2	3	1	3	1
**Trial 14**	2	4	2	1	4	2	2	3	1	4	4	1	2	C	4	3
**Trial 15**	4	1	4	3	C	1	1	C	3	C	C	4	4	2	C	2
**Trial 16**	3	3	3	2	3	4	4	2	1	C	3	1	C	4	1	4
**Trial 17**	4	C	C	3	C	1	C	C	2	2	C	C	4	3	4	1
**Trial 18**	C	1	2	C	4	C	3	1	C	1	2	3	3	2	C	3
**Trial 19**	2	2	4	4	2	3	1	3	3	3	4	2	2	1	2	C
**Trial 20**	1	4	1	1	1	2	2	4	4	4	1	4	1	C	3	2
**Trial 21**	2	2	2	3	3	3	3	1	1	1	1	3	1	C	C	3
**Trial 22**	3	4	1	2	1	2	4	4	3	4	3	1	2	4	2	2
**Trial 23**	C	3	3	C	2	4	2	C	4	3	4	C	3	2	4	C
**Trial 24**	4	C	C	4	4	C	C	2	2	2	C	4	C	3	3	4
**Trial 25**	1	1	4	1	C	1	1	3	C	C	2	2	4	1	1	1
**Trial 26**	1	1	1	3	C	4	3	1	4	4	1	2	1	C	C	3
**Trial 27**	2	2	2	2	3	C	1	3	3	3	2	1	2	4	2	C
**Trial 28**	4	4	C	1	4	3	2	C	C	1	3	3	3	3	3	4
**Trial 29**	C	3	3	C	1	2	4	2	1	C	C	4	4	2	4	1
**Trial 30**	3	C	4	4	2	1	C	4	2	2	4	C	C	1	1	2
**Trial 31**	C	C	4	C	C	1	2	C	2	3	4	4	C	4	3	1
**Trial 32**	1	1	1	2	4	4	1	3	4	4	1	C	4	2	4	3
**Trial 33**	4	2	3	3	2	3	4	2	3	1	2	3	3	3	C	2
**Trial 34**	2	4	C	1	3	2	C	4	1	2	C	1	2	1	1	C
**Trial 35**	3	3	2	4	1	C	3	1	C	C	3	2	1	C	2	4
**Trial 36**	1	1	1	3	C	C	C	2	4	2	1	1	3	2	2	C
**Trial 37**	3	3	4	1	4	3	4	4	1	4	4	3	2	C	4	3
**Trial 38**	C	C	3	4	1	2	3	1	3	1	C	C	4	4	3	4
**Trial 39**	2	4	C	C	3	4	2	C	C	3	3	2	1	3	C	1
**Trial 40**	4	2	2	2	2	1	1	3	2	C	2	4	C	1	1	2
**Trial 41**	3	3	C	3	2	4	1	4	3	C	2	1	4	3	C	3
**Trial 42**	2	1	4	1	1	C	2	2	C	1	C	C	C	1	1	4
**Trial 43**	4	4	1	2	4	3	C	1	2	2	3	4	2	C	3	2
**Trial 44**	C	2	3	4	C	1	3	3	1	4	1	2	3	2	2	1
**Trial 45**	1	C	2	C	3	2	4	C	4	3	4	3	1	4	4	C
**Trial 46**	C	C	4	3	2	C	1	3	2	2	4	3	4	3	2	C
**Trial 47**	3	3	1	C	C	4	4	C	4	C	C	C	3	2	4	3
**Trial 48**	1	1	C	4	4	2	2	1	C	1	2	2	2	4	3	1
**Trial 49**	2	4	2	2	3	1	3	2	3	3	1	4	C	C	1	4
**Trial 50**	4	2	3	1	1	3	C	4	1	4	3	1	1	1	C	2
